# Synthesis, Characterization and Microwave-Promoted Catalytic Activity of Novel *N*-phenylbenzimidazolium Salts in Heck-Mizoroki and Suzuki-Miyaura Cross-Coupling Reactions under Mild Conditions

**DOI:** 10.3390/molecules18032501

**Published:** 2013-02-25

**Authors:** Ülkü Yılmaz, Hasan Küçükbay, Selma Deniz, Nihat Şireci

**Affiliations:** 1 Battalgazi Vocational School, İnönü University, Battalgazi, 44210 Malatya, Turkey; E-Mail: ulku.yilmaz@inonu.edu.tr; 2 Department of Chemistry, Faculty of Science, İnönü University, 44280 Malatya, Turkey; 3 Department of Elementary Education, Faculty of Education, Hakkari University, 30000 Hakkari, Turkey; E-Mail: selmadeniz@hakkari.edu.tr; 4 Department of Elementary Education, Faculty of Education, Adıyaman University, 02040 Adıyaman, Turkey; E-Mail: nsireci@adiyaman.edu.tr

**Keywords:** *N*-phenylbenzimidazoles, Suzuki-Miyaura reaction, Heck-Mizoroki reaction, microwaves, NHC-precursor

## Abstract

A number of novel benzimidazolium salts having aryl substituents such as *N*-phenyl, 4-chlorophenyl and various alkyl substituents were synthesized. Their microwave-assisted catalytic activities were evaluated in Heck-Mizoroki and Suzuki-Miyaura cross-coupling reactions using a catalytic system consisting of Pd(OAc)_2_/K_2_CO_3_ in DMF/H_2_O under mild reaction conditions with consistent high yields, except those of 2-bromopyridine.

## 1. Introduction

In recent years, palladium catalyzed the C–C bond forming reaction of aryl halides with phenylboronic acid and styrene has been great interest for the synthesis of agrochemicals, pharmaceuticals and advanced enantioselective synthesis of natural products [1,2]. These ingenious carbon-carbon bond formation methods, the Heck-Mizoroki and Suzuki-Miyaura methods, also play vital roles in the development of new generation organic materials with novel electronic, optical or mechanical properties [3]. The Heck-Mizoroki and Suzuki-Miyaura reactions are most commonly used as model reactions to evaluate and compare the catalytic activity of metal complexes, to highlight ligand effects, and to understand the role of solvent and other additives [4,5,6,7,8]. Nevertheless, most efficient Heck-Mizoroki and Suzuki-Miyaura reactions reported were carried out in organic solvents with phosphine-based compounds as the ancillary ligand. In order to overcome the toxicity, air and/or moisture-sensitivity of phosphine-based ligands, many efforts have been made to find out alternative ligands [9,10,11,12]. In this context, *N*-heterocyclic carbenes (NHCs) are promising alternative ligands in palladium-catalyzed cross-coupling reactions due to their strong σ-donor but poor π-acceptor abilities, low toxicity, stability to air, moisture and heating. They are considered alternatives to phosphine ligands in metal complexes. For this reason, the design and synthesis of novel and effective NHCs have attracted a great deal of attention from both academia and industry [13,14,15,16,17]. Furthermore, ligand-free palladium catalyzed C–C coupling reactions were also studied in water [18,19,20,21,22].

On the other hand, the application of microwave irradiation for promoting reactions has become a versatile tool in organic synthesis. Rapid heating, reduced reaction times, and in many cases, increased yields have made microwave assisted organic synthesis a commonly used tool, especially for preparative medicinal chemistry. The shortened reaction times offered by microwave heating indicate that additional benefits could be obtained from an energy-saving perspective by exploiting this technology even further [23,24,25]. For this reason, microwave-promoted synthesis is an area of increasing interest in both academic and industrial laboratories. The use of metal catalysts in conjunction with microwaves may have significant advantages over traditional heating methods since the inverted temperature gradient under microwave conditions may lead to an increased lifetime of catalyst through the elimination of wall effects [26]. There are extensive studies about Heck-Mizoroki and Suzuki-Miyaura type C–C cross-coupling reactions incorporating microwave irradiation using various ligands other than the benzimidazole moiety [22,27,28,29,30,31,32,33,34,35].

During recent decades, many researchers have focused mainly on the development of milder reaction conditions using new catalysts and reaction systems. In order to find a more efficient palladium catalyst, we also synthesized a series of some new benzimidazole salts, containing alkyl or heterocycle substituted alkyl and bis-benzimidazole salts as a NHC ligand and we tested the activity of Pd-NHC based catalytic systems prepared *in-situ* for the Heck-Mizoroki and Suzuki-Miyaura cross-coupling reactions under microwave heating conditions [36,37,38,39,40].

In continuation of this subject, herein we describe the synthesis of new benzimidazole salts **1**–**10** containing phenyl or 4-chlorophenyl on one nitrogen atom of the benzimidazole ring and an alkyl or substituted alkyl on the other nitrogen atom. The compounds were fully characterized by elemental analysis, IR, ^13^C-NMR, and ^1^H-NMR spectroscopy. We also report the microwave-assisted catalytic activity of Pd(OAc)_2_/base/novel benzimidazoles catalytic system in Heck-Mizoroki and Suzuki-Miyaura cross-coupling reactions.

## 2. Results and Discussion

1-Phenylbenzimidazole and 1-(4-chlorophenyl)benzimidazole were synthesized from *N*-phenyl-1,2-diaminobenzene and *N*-(4-chlorophenyl)-1,2-diaminobenzene by reaction with formic acid in 4N HCl according to the Phillips’ method, respectively [41,42].

**Scheme 1:**
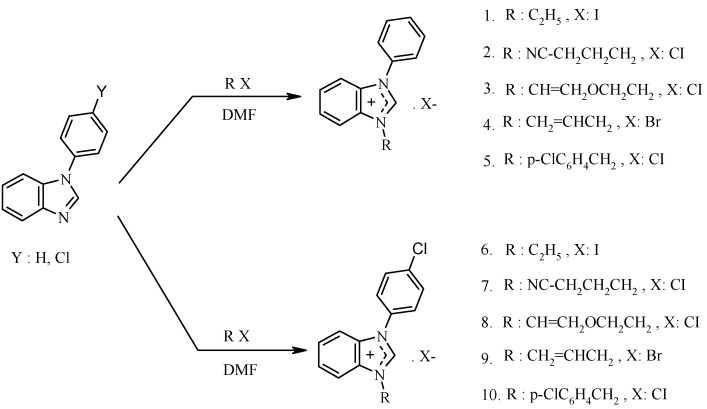
Synthesis of *N*-arylbenzimidazole derivatives.

Novel benzimidazolium salts, **1**–**10** were prepared in good yields of 70%–92% by treatment of 1-phenylbenzimidazole or 1-(4-chlorophenyl)benzimidazole with appropriate alkyl halides in refluxing DMF. The synthesis of the benzimidazolium salts **1**–**10** is summarized in Scheme 1.

**Scheme 2:**
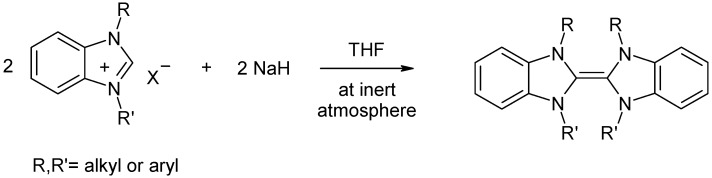
Electron-rich olefin synthesis through the acidic hydrogen atom removing on the 2-position of the benzimidazole.

The benzimidazolium salts are air and moisture-stable both in the solid state and in solution. The new benzimidazole derivatives **1**–**10** were characterized by ^1^H-NMR, ^13^C {^1^H} NMR, IR and elemental analysis techniques, which support the proposed structures. The value of δ[^13^C{^1^H}], N*C*H_2_Ph in benzimidazolium salts **5** and **10** were both found to be 49.9 ppm. The benzylic protons signals for the benzimidazolium salts **5** and **10** were found to be 5.89 and 5.92 ppm, respectively. The value of δ[^13^C{^1^H}], N*C*HN in benzimidazolium salts is usually around 142 ± 4 [37]. For benzimidazolium salts, **1**–**10** it was found to be 142.8, 143.6, 143.3, 143.3, 143.6, 142.9, 143.7, 143.5, 143.4 and 143.7 ppm, respectively. These values are in good agreement with the previously reported results [10]. The NC*H*N proton signals for the benzimidazolium salts, **1**–**10** were observed as singlets at 10.20, 10.74, 10.32, 10.28, 10.51, 10.18, 10.51, 10.30, 10.34 and 10.67 ppm, respectively. As expected, the highest downfield shifts of the NC*H*N proton signals were observed among the benzimidazolium salts which bear electron withdrawing chloride and cyanide substituents on the nitrogen atom of the benzimidazole scaffold. Thus, the hydrogen atom on the 2-position of the benzimidazolium salts behave as acids in the sense that they give up protons to suitably strong bases. As a result of acidic proton of these type benzimidazolium salts, electron-rich olefins can be synthesized easily, as shown in Scheme 2 [43,44,45]. The carbon-nitrogen band frequencies, ν_(C=N)_ for benzimidazole salts, **1**–**10** were observed at 1560, 1557, 1558, 1554 and 1558, 1559, 1557, 1557, 1552 and 1554 cm^−1^, respectively. 

### 2.1. The Heck-Mizoroki Coupling Reaction

The Heck-Mizoroki coupling is one of the most studied C–C bond forming reactions between alkenes and aromatic rings and is widely used by both academic and industrial laboratories. The industrial applications of this reaction can be observed in the fine chemical field, such as in the manufacture of pharmaceuticals and herbicides [46,47,48]. The catalytic yield of the Heck-Mizoroki coupling reaction is dependent on a variety of parameters such as temperature, solvent, base and nature of the catalyst and catalyst loading. For this reason, the optimum reaction parameters were investigated before starting the series of coupling experiments.

In order to find the optimum reaction conditions for the Heck-Mizoroki coupling reaction, a series of experiments was performed with 2-bromopyridine and styrene as model compounds. The test reactions were performed using different bases such as K_2_CO_3_, Cs_2_CO_3_, Et_3_N and DBU (1,8-diazabicyclo[5.4.0]undec-7-ene) and different solvents such as DMF/H_2_O, EtOH/H_2_O, DMA and C_2_H_4_(OH)_2_/H_2_O for different times and temperatures. It was found that the Heck-Mizoroki coupling reaction catalyzed by **1**, Pb(OAc)_2_ and base catalyst system gave the highest yield when using DMF/H_2_O mixture as a solvent and K_2_CO_3_ or Cs_2_CO_3_ as a base at 80 and 100 °C microwave heating in 10 min. Very little improvement was observed for the catalytic yields upon increasing the temperature from 80 °C to 100 °C. No considerable increase in catalytic reaction yields was observed by prolonging the time from 10 to 20 min either. After these results, we chose K_2_CO_3_ as a base, being cheaper than Cs_2_CO_3_, water/DMF as a solvent and 80 °C as reaction temperature for the lower energy consumption than at 100 °C. We also tested the catalytic yields using a conventional heating system in a preheated oil bath 5, 10 and 20 min at 60 °C and 80 °C, but the yields of the catalytic conversions were either nil or very low (Table 1, entries 1–6). Control experiments showed that the yield of Heck-Mizoroki coupling reaction were decreased in the absence of **1** in 10 min under microwave heating (Table 1, entries 16, 17). Use of a higher amounts of benzimidazolium salt (4 mol %) did not improve the catalytic yield (Table 1, entry 18). The coupling reaction did not occur in the absence of Pd(OAc)_2_ after 10 min under microwave heating (Table 1, entry 19). The test experiments results for optimization of the Heck-Mizoroki coupling reaction are given in Table 1. After optimization experiments (Table 1), we found that use of the catalytic system consists of 1% mol Pd(OAc)_2_, 2% mol of **1**–**10** and 2 mol K_2_CO_3_ in DMF/H_2_O (1:1) at 80 °C/300 W microwave heating led to the best conversion within 10 min.

**Table 1:** Test experiments for optimization of the Heck-Mizoroki coupling reactions.

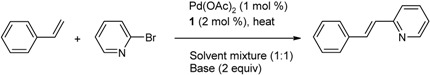								
Entry	Ligand	Base	Solvent	Time (min)	Thermal	heating	Microwave	heating
					°C	Yield,%	°C (300 W)	Yield,%
1	**1**	K_2_CO_3_	DMF/H_2_O	5	60	n.d.	60	33
2	**1**	K_2_CO_3_	DMF/H_2_O	10	60	n.d.	60	49
3	**1**	K_2_CO_3_	DMF/H_2_O	20	60	17	60	52
4	**1**	K_2_CO_3_	DMF/H_2_O	5	80	03	80	46
5	**1**	K_2_CO_3_	DMF/H_2_O	10	80	09	80	63
6	**1**	K_2_CO_3_	DMF/H_2_O	20	80	13	80	64
7	**1**	K_2_CO_3_	DMF/H_2_O	10			100	65
8	**1**	CsCO_3_	DMF/H_2_O	10			80	62
9	**1**	CsCO_3_	EtOH/H_2_O	10			80	43
10	**1**	Et_3_N	DMF/H_2_O	10			80	57
11	**1**	Et_3_N	EtOH/H_2_O	10			80	52
12	**1**	DBU	DMF/H_2_O	10			80	59
13	**1**	DBU	EtOH/H_2_O	10			80	54
14	**1**	K_2_CO_3_	C_2_H_4_(OH)_2_/H_2_O	10			80	51
15	**1**	K_2_CO_3_	DMA	10			80	32
16	**no**	K_2_CO_3_	DMF/H_2_O	10			80	16
17	**no**	DBU	EtOH/H_2_O	10			80	13
18	**1**	K_2_CO_3_	DMF/H_2_O	10			80	63 ^k^
19	**1**	K_2_CO_3_	DMF/H_2_O	10			80	n.d. ^l^

n.d.: not detected. Reaction conditions are same as indicated in the text. Yields are based on aryl bromide. Reactions were monitored by GC-MS. 4 mol % of 1 ^k^, without Pd(OAc)_2_^l^.

Using the above optimized reaction conditions, the coupling reactions of four different aryl halides (bearing electron-donating, electron-withdrawing groups and 2-bromopyridine) and styrene were then investigated. The results are shown in Table 2. Among the aryl halides, the catalytic yield of those involving 2-bromopyridine were found to be moderate but the others gave high yields. 

**Table 2:** The Heck-Mizoroki coupling reactions of aryl halides with styrene.

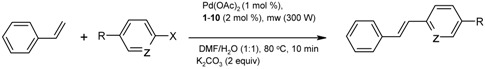					
Entry	R	Z	X	Salt	Conversion ^a^ (%)
1	H	N	Br	1	46 ^b^
2	H	N	Br	1	63 ^c^
3	H	N	Br	1	64 ^d^
4	H	N	Br	1	03 ^e^
5	H	N	Br	1	09 ^f^
6	H	N	Br	no	16 ^g^
7	H	CH	I	1	98
8	H	CH	I	2	98
9	H	CH	I	3	97
10	H	CH	I	4	96
11	H	CH	I	5	96
12	H	CH	I	6	93 91 ^i^
13	H	CH	I	7	90 ^i^
14	H	CH	I	8	89 ^i^
15	H	CH	I	9	87 ^i^
16	H	CH	I	10	88 ^i^
17	OCH_3_	CH	I	1	99
18	OCH_3_	CH	I	2	98
19	OCH_3_	CH	I	3	98
20	OCH_3_	CH	I	4	98
21	OCH_3_	CH	I	5	97
22	OCH_3_	CH	I	6	95^i^
23	OCH_3_	CH	I	7	94 89 ^i^
24	OCH_3_	CH	I	8	90 ^i^
25	OCH_3_	CH	I	9	91 ^i^
26	OCH_3_	CH	I	10	90^i^
27	COCH_3_	CH	Br	1	99
28	COCH_3_	CH	Br	2	99
29	COCH_3_	CH	Br	3	98
30	COCH_3_	CH	Br	4	95
31	COCH_3_	CH	Br	5	96
32	COCH_3_	CH	Br	6	94 ^i^
33	COCH_3_	CH	Br	7	96 92 ^i^
34	COCH_3_	CH	Br	8	90 ^i^
35	COCH_3_	CH	Br	9	87 ^i^
36	COCH_3_	CH	Br	10	86 ^i^
37	H	N	Br	2	62
38	H	N	Br	3	62
39	H	N	Br	4	60
40	H	N	Br	5	61
41	H	N	Br	6	59
42	H	N	Br	7	58
43	H	N	Br	8	56
44	H	N	Br	9	57
45	H	N	Br	10	58

^a^ Conversions were determined by GC-MS based on the aryl halide. Reaction conditions: temperature ramped to 80 °C (3 min) and held for 5 ^b^ min, 10 ^c^ min and 20 ^d^ min. In preheated oil bath, 5 ^e^ min and 10 ^f^ min with thermal heating at 80 °C. Temperature ramped to 80 °C (3 min) and held for 10 ^g^ min without salt (**1**). Isolated yields; ^i^ n.d., not detected.

The benzimidazolium salts bearing an electron releasing ethyl substituent (**1** and **6**) are generally more effective than other salts examined. On the other hand, benzimidazole salts having a 4-chlorophenyl substituent on the nitrogen atom were generally slightly less reactive for the Heck-Mizoroki reaction than the corresponding phenyl substituent. Of the four different aryl halides used in the Heck-Mizoroki coupling with styrene, those with electron-withdrawing substituents (4-bromoacetphenone) were found to give the highest yield (Table 2, entries 27–36). Furthermore, aryl iodides, those with electron releasing methoxy substituents and without any substituent were also found to give better yield than 2-bromopyridine.

### 2.2. The Suzuki-Miyaura Coupling Reaction

Biaryls represent the mostly common structural motif in a broad range of susbtances, from naturally occurring potentially useful therapeutic agents to versatile building materials for light-emitting diodes, liquid crystals and many organic compounds with novel electronic, optical properties [49,50].

In order to find the optimum reaction conditions for the Suzuki coupling reaction, a series of experiments catalyzed couplings between 2-bromopyridine and phenylboronic acid as a model compounds were performed using similar parameters as in the Heck-Mizoroki coupling reactions described above. It was found that the Suzuki-Miyaura coupling reaction catalyzed by **1**, Pb(OAc)_2_ and base catalyst system gave the highest yield when using DMF/H_2_O mixture as a solvent and K_2_CO_3_ or Cs_2_CO_3_ as a base at 80 °C microwave heating in 10 min. After these results, we chose K_2_CO_3_ as a base, being cheaper than Cs_2_CO_3_, water/DMF as a solvent and 80 °C as reaction temperature for the optimum reaction conditions. The conventional heating system in a preheated oil bath 5, 10 and 20 min at 60 °C, 80 °C and 100 ° C was not appropriate for these conversions (Table 3, entries 1–9). When both microwave results and conventional preheated oil bath results were compared, we observed a clear improvement in yield and reaction time with microwave heating. Control experiments showed that the yield of Suzuki coupling reaction was decreased in the absence of **1** in 10 min under microwave heating (Table 3, entry 18). No coupling reaction was observed in the absence of Pd(OAc)_2_ after 10 min under microwave heating (Table 3, entry 20). The test experiments results for optimization of the Suzuki-Miyaura coupling reaction are given in Table 3.

After having established the optimized coupling reaction conditions (Table 3) the scope of the reaction and efficiencies of the benzimidazolium salts were evaluated by investigating the coupling of the phenylboronic acid with various aryl halides and a heteroaryl bromide. Under the optimized conditions, reaction of *p*-bromoacetophenone, *p*-iodoanisole and iodobenzene with phenylboronic acid gave almost identical high yields using a catalytic system consisting of 2 mol % benzimidazole salt **1**–**10**, 1 mol % Pd(OAc)_2_ and 2 equiv. K_2_CO_3_ in DMF/H_2_O (1:1) at 80 °C under microwave irradiation (300 W) within 10 min. On the other hand, 2-bromopyridine gave a moderate yield using the optimized conditions. This is also good result considering the difficult synthesis of 2-arylpyridines using expensive organometallic reagents such as phenyl lithium or phenyl magnesium halides under an inert atmosphere. Of the four different aryl halides used in the Suzuki-Miyaura coupling with phenylboronic acid, those with electron-withdrawing substituents were found to give the highest yield (Table 4, entries 27–36). 

**Table 3:** Test experiments for optimization of the Suzuki-Miyaura coupling reactions.

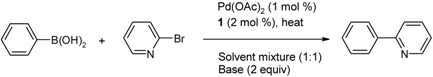								
Entry	Ligand	Base	Solvent	Time (min)	Thermal	heating	Microwave	heating
					°C	Yield,%	°C (300 W)	Conver. ^a^, %
1	**1**	K_2_CO_3_	DMF/H_2_O	5	60	0	60	47
2	**1**	K_2_CO_3_	DMF/H_2_O	10	60	4	60	53
3	**1**	K_2_CO_3_	DMF/H_2_O	20	60	9	60	55
4	**1**	K_2_CO_3_	DMF/H_2_O	5	80	6	80	67
5	**1**	K_2_CO_3_	DMF/H_2_O	10	80	11	80	75
6	**1**	K_2_CO_3_	DMF/H_2_O	20	80	13	80	76
7	**1**	K_2_CO_3_	DMF/H_2_O	5	100	7	100	67
8	**1**	K_2_CO_3_	DMF/H_2_O	10	100	11	100	76
9	**1**	K_2_CO_3_	DMF/H_2_O	20	100	14	100	77
10	**1**	CsCO_3_	DMF/H_2_O	10			80	75
11	**1**	CsCO_3_	EtOH/H_2_O	10			80	66
12	**1**	K_2_CO_3_	H_2_O	10			80	39
13	**1**	K_2_CO_3_	C_2_H_4_(OH)_2_/H_2_O	10			80	56
14	**1**	K_2_CO_3_	DMA	10			80	47
15	**1**	DBU	DMF/H_2_O	10			80	64
16	**1**	DBU	EtOH/H_2_O	10			80	66
17	**1**	K_2_CO_3_	Glycerine/H_2_O	10			80	58
18	**no**	K_2_CO_3_	DMF/H_2_O	10			10	32
19	**1**	K_2_CO_3_	DMF/H_2_O	10			10	74 ^m^
20	**1**	K_2_CO_3_	DMF/H_2_O	10			10	n.d. ^n^
21	**1**	K_2_CO_3_	DMF/H_2_O	10			10	77 ^p^
22	**1**	K_2_CO_3_	DMF/H_2_O	10			10	65 ^r^

^a^ Conversions were determined by GC-MS based on the aryl halide. 4 mol % of 1 ^m^, without Pd(OAc)_2_^n^, 2 mol % of Pd(OAc)_2_^p^, 0.5 mol % of Pd(OAc)_2_^r^.

**Table 4:** The Suzuki-Miyaura coupling reactions of aryl halides with phenylboronic acid.

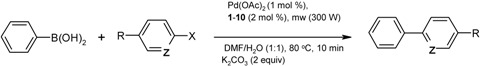					
Entry	R	Z	X	Salt	Conversion ^a^ (%)
1	H	N	Br	1	67 ^b^
2	H	N	Br	1	75 ^c^
3	H	N	Br	1	76 ^d^
4	H	N	Br	1	06 ^e^
5	H	N	Br	1	11 ^f^
6	H	N	Br	no	32 ^g^
7	H	CH	I	1	98
8	H	CH	I	2	98
9	H	CH	I	3	97
10	H	CH	I	4	97
11	H	CH	I	5	96
12	H	CH	I	6	97 95 ^i^
13	H	CH	I	7	94^i^
14	H	CH	I	8	91^i^
15	H	CH	I	9	90^i^
16	H	CH	I	10	90^i^
17	OCH_3_	CH	I	1	99
18	OCH_3_	CH	I	2	99
19	OCH_3_	CH	I	3	97
20	OCH_3_	CH	I	4	98
21	OCH_3_	CH	I	5	98
22	OCH_3_	CH	I	6	97 95 ^i^
23	OCH_3_	CH	I	7	95 ^i^
24	OCH_3_	CH	I	8	94 ^i^
25	OCH_3_	CH	I	9	94 ^i^
26	OCH_3_	CH	I	10	93 ^i^
27	COCH_3_	CH	Br	1	99
28	COCH_3_	CH	Br	2	99
29	COCH_3_	CH	Br	3	99
30	COCH_3_	CH	Br	4	99
31	COCH_3_	CH	Br	5	98
32	COCH_3_	CH	Br	6	98 96 ^i^
33	COCH_3_	CH	Br	7	96 ^i^
34	COCH_3_	CH	Br	8	95 ^i^
35	COCH_3_	CH	Br	9	95 ^i^
36	COCH_3_	CH	Br	10	95 ^i^
37	H	N	Br	2	72
38	H	N	Br	3	69
39	H	N	Br	4	70
40	H	N	Br	5	63
41	H	N	Br	6	70
42	H	N	Br	7	67
43	H	N	Br	8	68
44	H	N	Br	9	70
45	H	N	Br	10	70

^a^ Conversions were determined by GC-MS based on the aryl halide. Reaction conditions: temperature ramped to 80 °C (3 min) and held for 5 ^b^ min, 10 ^c^ min and 20 ^d^ min. In preheated oil bath, 5 ^e^ min and 10 ^f^ min with thermal heating at 80 °C. Temperature ramped to 80 °C (3 min) and held for 10 ^g^ min without salt (**1**). Isolated yields ^i^.

The benzimidazole salts bearing an electron-withdrawing chloro substituent on the *para*-position of the phenyl ring, **6**–**10**, were found to be the less effective of the salts examined in the Suzuki-Miyaura cross-coupling reactions (Table 4, entries 12–16, 22–26, 32–36 and 41–45). On the other hand, benzimidazole salts **1** and **6** bearing an electron-donating ethyl group on the nitrogen atom are the most effective for the catalytic activity in Suzuki coupling reactions. Similar catalytic results for the Suzuki-Miyaura cross-coupling reactions have also been obtained using Pd(OAc)_2_ or PdCl_2_, base and benzimidazole or imidazole catalytic systems bearing different electron-donating or electron-withdrawing aryl, substituted aryl, alkyl and substituted alkyl groups on benzimidazole or imidazole ligands [51,52,53]. A comparison of our catalytic system consisting of Pd(OAc)_2_/benzimidazolium salt/K_2_CO_3_ in DMF/H_2_O under microwave heating with similar catalytic systems including NHC ligand [8,14,54,55,56], under conventional heating clearly indicates that microwave heating improves in catalytic yields and reaction times.

## 3. Experimental

### 3.1. General Chemical Procedure

Starting materials and reagents used were of commercial grade and purchased from Aldrich or Merck Chemical Co. Solvents were dried with standard methods and freshly distilled prior to use. All catalytic activity experiments were carried out in a microwave oven manufactured by Milestone (Milestone Start S Microwave Labstation for Synthesis, Sorisole, Italy) under aerobic conditions. ^1^H-NMR (300 MHz) and ^13^C-NMR (75 MHz) spectra were recorded using a Bruker DPX-300 high performance digital FT NMR spectrometer (Billercia, MA, USA). Infrared spectra were recorded from KBr pellets in the range 4000–400 cm^−1^ on a Perkin-Elmer FT-IR spectrophotometer. Elemental analyses were performed with a LECO CHNS-932 elemental analyzer (St. Joseph, MI, USA). Melting points were recorded using an electrothermal-9200 melting point apparatus, and are given uncorrected.

*Synthesis of 3-ethyl-1-phenylbenzimidazolium iodide* (**1**). To a solution of 1-phenylbenzimidazole (1.35 g, 6.95 mmol) in dimethylformamide (5 mL) was added ethyl iodide (0.60 cm^3^, 6.95 mmol) and the mixture was heated under reflux for 3 h. The mixture was then cooled and the solvent was removed in vacuo. The residue was crystallized from EtOH/Et_2_O (1:1). Yield 1.90 g, 78%; m.p.: 203–204 °C; υ _(N=C)_: 1560 cm^−1^. Anal. found: C, 51.32; H, 4.29; N, 7.94%. Calcd for C_15_H_15_N_2_I: C, 51.45; H, 4.32; N, 8.00%. ^1^H-NMR (300.13 MHz, DMSO-d_6_): δ 10.20 (s, 1H, NCHN), 8.24 (d, 1H, C_6_H_4_, *J* = 7.5 Hz), 7.86 (m, 3H, C_6_H_4_), 7.78–7.70 (m, 5H, C_6_H_5_), 4.63 (q, 2H, CH_2_CH_3_, *J* = 7.2 Hz), 1.64 (t, 3H, CH_2_CH_3_, *J* = 7.2 Hz). ^13^C-NMR (75.47 MHz, DMSO-d_6_): δ 142.8 (NCHN), 133.7, 131.6, 131.5, 130.9, 130.8, 127.9, 127.4, 125.7, 114.5, 113.9 (C_6_H_4_ and C_6_H_5_), 42.9 (CH_2_CH_3_), 14.5 (CH_2_CH_3_).

*Synthesis of 3-(3-cyanopropyl)-1-phenylbenzimidazolium chloride* (**2**). This compound was similarly was synthesized from 1-phenylbenzimidazole and 4-chloro-butyronitrile. Yield 1.54 g, 74%; m.p.: 101–102 °C; υ _(N=C)_: 1557 cm^−1^. Anal. found: C, 68.44; H, 5.35; N, 14.06%. Calcd for C_17_H_16_N_3_Cl: C, 68.57; H, 5.42; N, 14.11%. ^1^H-NMR (300.13 MHz, DMSO-d_6_): δ 10.74 (s, 1H, NCHN), 8.35 (d, 1H, C_6_H_4_, *J* = 8.1 Hz), 7.91–7.84 (m, 3H, C_6_H_4_), 7.79–7.68 (m, 5H, C_6_H_5_), 4.81 (t, 2H, CH_2_CH_2_CH_2_CN, *J* = 6.6 Hz), 2.86 (t, 2H, CH_2_CH_2_CH_2_CN, *J* = 7.2 Hz), 2.40 (m, 2H, CH_2_CH_2_CH_2_CN). ^13^C-NMR (75.47 MHz, DMSO-d_6_): δ 143.6 (NCHN), 133.7, 131.7, 131.4, 130.8, 127.9, 127.4, 125.6, 120.5, 114.6, 113.9 (C_6_H_4_ and C_6_H_5_), 46.3 (CH_2_CH_2_CH_2_CN), 34.3 (CH_2_CH_2_CH_2_CN), 25.0 (CH_2_CH_2_CH_2_CN), 14.3 (CH_2_CH_2_CH_2_CN).

*Synthesis of 3-(2-vinyloxy)ethyl-1-phenylbenzimidazolium chloride* (**3**). Synthesized from 1-phenylbenz-imidazole and 2-chloroethyl vinyl ether. Yield 1.66 g, 79%; m.p.: 112–113 °C; υ _(N=C)_: 1558 cm^−1^. Anal. found: C, 67.76; H, 5.64; N, 9.22%. Calcd for C_17_H_17_N_2_OCl: C, 67.88; H, 5.70; N, 9.31%. ^1^H-NMR (300.13 MHz, DMSO-d_6_): δ 10.32 (s, 1H, NCHN), 8.25 (d, 1H, C_6_H_4_, *J* = 7.8 Hz), 7.90–7.87 (m, 3H, C6H4), 7.80–7.70 (m, 5H, C_6_H_5_), 5.52 (t, 1H, CH_2_CH_2_OCH=CH_2_, *J* = 5.7 Hz), 4.69 (t, 2H, CH_2_CH_2_OCH=CH_2_, *J* = 4.6 Hz), 3.92 (two d, 2H, CH_2_CH_2_OCH=CHH, *J* = 4.8 Hz and 15 Hz), 3.40 (t, 2H, CH_2_CH_2_OCH=CH_2_, *J* = 4.6 Hz). ^13^C-NMR (75.47 MHz, DMSO-d_6_): δ 143.3 (NCHN), 143.4, 133.7, 132.1, 131.5, 130.9, 130.8, 127.7, 127.2, 125.7 (C_6_H_4_ and C_6_H_5_), 114.9, 113.8 (CH=CH_2_), 59.2 (CH_2_CH_2_OCH=CH_2_), 50.4 (CH_2_CH_2_OCH=CH_2_).

*Synthesis of 3-allyl-1-phenylbenzimidazolium bromide* (**4**). Synthesized from 1-phenylbenzimidazole and allyl bromide. Yield 1.87 g, 85%; m.p.: 171–172 °C; υ _(N=C)_: 1554 cm^−1^. Anal. found: C, 60.91; H, 4.73; N, 8.73%. Calcd for C_16_H_15_N_2_Br: C, 60.97; H, 4.80; N, 8.89%. ^1^H-NMR (300.13 MHz, DMSO-d_6_): δ 10.28 (s, 1H, NCHN), 8.40 (d, 1H, C_6_H_4_, *J* = 7.2 Hz), 7.96–7.85 (m, 3H, C6H4, 7.82–7.77 (m, 5H, C_6_H_5_), 6.26–6.13 (m, 1H, CH_2_CH=CH_2_), 5.60 (dd, 1H, CH_2_CH=CHH, *J* = 15.9; 1.2 Hz), 5.46 (dd, 1H, CH_2_CH=CHH, *J* = 9.0; 1.2 Hz), 5.30 (d, 2H, CH_2_CH=CH_2_, *J* = 6.0 Hz). ^13^C-NMR (75.47 MHz, DMSO-d_6_): δ 143.3 (NCHN), 133.6, 131.7, 131.5, 131.2, 130.9, 130.8, 127.9, 127.4, 125.7, 121.4 (C_6_H_4_ and C_6_H_5_), 114.7, 114.1 (CH_2_CH=CH_2_), 49.7 (CH_2_CH=CH_2_).

*Synthesis of 3-p-chlorobenzyl-1-phenylbenzimidazolium chloride* (**5**). Synthesized from 1-phenylbenz-imidazole and p-chlorobenzyl chloride. Yield 1.88 g, 76%; m.p.: 82–83 °C; υ _(N=C)_: 1558 cm^−1^. Anal. found: C, 67.45; H, 4.51; N, 7.73%. Calcd for C_20_H_16_N_2_Cl_2_: C, 67.62; H, 4.54; N, 7.89%. ^1^H-NMR (300.13 MHz, DMSO-d_6_): δ 10.51 (s, 1H, NCHN), 8.06–8.03 (m, 1H, C_6_H_4_), 7.91–7.86 (m, 3H, C_6_H_4_), 7.76–7.73 (m, 5H, C_6_H_5_), 7.71 (d, 2H, C_6_H_4_, *J* = 8.4 Hz), 5.89 (s, 2H, CH_2_). ^13^C-NMR (75.47 MHz, DMSO-d_6_): δ 143.6 (NCHN), 134.0, 133.6, 133.1, 131.8, 131.3, 131.0, 130.8, 129.4, 128.0, 127.6, 125.8, 114.6, 114.2 (C_6_H_4_ and C_6_H_5_), 49.9 (NCH_2_-).

*Synthesis of 3-Ethyl-1-p-chlorophenylbenzimidazolium iodide* (**6**). To a solution of 1-*p*-chlorophenylbenz-imidazole (1.37g, 6.0 mmol) in dimethylformamide (5 mL) was added ethyl iodide (0.50 cm^3^, 6.0 mmol) and the mixture was heated under reflux for 3 h. The mixture was then cooled and the solvent was removed in vacuo. The residue was crystallized from EtOH/Et_2_O (1:1). Yield 1.98 g, 86%; m.p.: 216–217 °C; υ _(N=C)_: 1559 cm^−1^. Anal. found: C, 46.56; H, 3.63; N, 7.09%. Calcd for C_15_H_14_N_2_ICl: C, 46.84; H, 3.67; N, 7.28%. ^1^H-NMR (300.13 MHz, DMSO-d_6_): δ 10.18 (s, 1H, NCHN), 8.23 (d, 1H, C_6_H_4_, *J* = 6.3 Hz), 7.92–7.71 (m, 7H, C_6_H_4_ and C_6_H_4_Cl), 4.62 (q, 2H, CH_2_CH_3_, *J* = 7.3 Hz), 1.63 (t, 3H, CH_2_CH_3_, *J* = 7.3 Hz). ^13^C-NMR (75.47 MHz, DMSO-d_6_): δ 142.9 (NCHN), 133.5, 132.6, 131.6, 131.4, 130.8, 127.9, 127.7, 127.4, 114.5, 113.9 (C_6_H_4_ and C_6_H_4_Cl), 42.9 (CH_2_CH_3_), 14.4 (CH_2_CH_3_).

*Synthesis of 3-(3-cyanopropyl)-1-p-chlorophenylbenzimidazolium chloride* (**7**). Similarly synthesized from 1-*p*-chlorophenylbenzimidazole and 4-chlorobutyronitrile. Yield 1.44 g, 72%; m.p.: 101–102 °C; υ _(N=C)_: 1557 cm^−1^. Anal. found: C, 61.14; H, 4.43; N, 12.18%. Calcd for C_17_H_15_N_3_Cl_2_: C, 61.46; H, 4.55; N, 12.65%. ^1^H-NMR (300.13 MHz, DMSO-d_6_): δ 10.51 (s, 1H, NCHN), 8.27 (d, 1H, C_6_H_4_, *J* = 8.1 Hz), 7.93–7.71 (m, 7H, C_6_H_4_ and C_6_H_4_Cl), 4.72 (t, 2H, CH_2_CH_2_CH_2_CN, *J* = 6.45 Hz), 2.81 (t, 2H, CH_2_CH_2_CH_2_CN, *J* = 7.2 Hz), 2.38 (m, 2H, CH_2_CH_2_CH_2_CN). ^13^C-NMR (75.47 MHz, DMSO-d_6_): δ 143.7 (NCHN), 135.5, 132.5, 131.6, 131.5, 130.9, 127.9, 127.7, 120.4, 114.5, 113.9 (C_6_H_4_ and C_6_H_4_Cl), 46.3 (CH_2_CH_2_CH_2_CN), 34.4 (CH_2_CH_2_CH_2_CN), 24.9 (CH_2_CH_2_CH_2_CN), 14.3 (CH_2_CH_2_CH_2_CN).

*Synthesis of 3-(2-vinyloxyethyl)-1-p-chlorophenylbenzimidazolium chloride* (**8**). Synthesized from 1-*p*-chlorophenylbenzimidazole and 2-chloroethyl vinyl ether. Yield 1.41 g, 70%; m.p.: 164–166 °C; υ _(N=C)_: 1557 cm^−1^. Anal. found: C, 60.26; H, 4.64; N, 8.08%. Calcd for C_17_H_16_N_2_OCl_2_: C, 60.91; H, 4.81; N, 8.36%. ^1^H-NMR (300.13 MHz, DMSO-d_6_): δ 10.30 (s, 1H, NCHN), 8.25 (d, 1H, C_6_H_4_, *J* = 8.4 Hz), 7.94–7.70 (m, 7H, C_6_H_4_ and C_6_H_4_Cl), 5.54 (t, 1H, CH_2_CH_2_OCH=CH_2_, *J* = 6.0 Hz), 4.68 (t, 2H, CH_2_CH_2_OCH=CH_2_, *J* = 4.6 Hz), 3.92 (dd, 2H, CH_2_CH_2_OCH=CHH, *J* = 5.0 Hz), 3.40 (t, CH_2_CH_2_CH_2_CN). ^13^C-NMR (75.47 MHz, DMSO-d_6_): δ 143.5 (NCHN), 133.5, 131.9, 131.6, 130.8, 127.8, 127.7, 127.3 (C_6_H_4_ and C_6_H_4_Cl), 114.8, 113.8 (CH=CH_2_), 59.1 (CH_2_CH_2_OCH=CH_2_), 50.5 (CH_2_CH_2_OCH=CH_2_).

*Synthesis of 3-Allyl-1-p-chlorophenylbenzimidazoliumBromide* (**9**). Synthesized from 1-p-chlorophenyl-benzimidazole and allyl bromide. Yield 1.93 g, 92%; m.p.: 119–121 °C; υ _(N=C)_: 1552 cm^−1^. Anal. found: C, 54.23; H, 4.03; N, 7.83%. Calcd for C_16_H_14_N_2_BrCl: C, 54.96; H, 4.04; N, 8.01%. ^1^H-NMR (300.13 MHz, DMSO-d_6_): δ 10.34 (s, 1H, NCHN), 8.14 (d, 1H, C_6_H_4_, *J* = 8.4 Hz), 7.94–7.70 (m, 7H, C**_6_**H_4_ and C_6_H_4_Cl), 6.26–6.14 (m, 1H, CH_2_CH=CH_2_), 5.60 (d, 1H, CH=CHH, *J* = 17.1 Hz), 5.46 (d, 1H, CH_2_CH=CHH, *J* = 10.2Hz), 5.32 (d, 2H, CH_2_CH=CH_2_, *J* = 5.7 Hz). ^13^C-NMR (75.47 MHz, DMSO-d_6_): δ 143.4 (NCHN), 135.5, 132.5, 131.7, 131.4, 131.1, 130.8, 127.9, 127.8, 127.4, 121.4 (C_6_H_4_ and C_6_H_4_Cl), 114.7, 114.0 (CH_2_CH=CH_2_), 49.7 (CH_2_CH=CH_2_).

*Synthesis of 3-p-Chlorobenzyl-1-p-chlorophenylbenzimidazoliumChloride* (**10**). Synthesized from 1-*p*-chlorophenylbenzimidazole and p-chlorobenzyl chloride. Yield 2.01 g, 86%; m.p.: 209–210 °C; υ _(N=C)_: 1554 cm^−1^. Anal. found: C, 61.05; H, 3.79; N, 7.02%. Calcd for C_20_H_15_N_2_Cl_3_: C, 61.64; H, 3.88; N, 7.19%. ^1^H-NMR (300.13 MHz, DMSO-d_6_): δ 10.67 (s, 1H, NCHN), 8.06–7.49 (m, 12H, C_6_H_4_, C_6_H_4_Cl and CH_2_C_6_H_4_Cl), 5.92 (s, 2H, CH_2_). ^13^C-NMR (75.47 MHz, DMSO-d_6_): δ 143.7 (NCHN), 135.5, 134.0, 133.5, 133.1, 132.5, 131.8, 131.1, 130.7, 129.3, 128.0, 127.7, 127.5, 114.6, 114.1 (C_6_H_4_, NC_6_H_4_Cl and NCH_2_C_6_H_4_Cl), 49.9 (NCH_2_).

### 3.2. General Procedure for the Heck-Mizoroki Reactions

Pd(OAc)_2_ (1 mmol %), benzimidazolium halides **1**–**10** (2 mmol %), the aryl halide (1 mmol), styrene (1.2 mmol), K_2_CO_3_ (2 mmol), water (3 mL), and DMF (3 mL) were added to a microwave apparatus and the mixture was heated at 80 °C (300 W) for 10 min. A ramp time of 3 min was used to reach the temperature of 80 °C. At the end of reaction, the mixture was cooled; the product was extracted with ethyl acetate/*n*-hexane (1:5) and filtered through a pad of silica gel with copious washing. The percent conversion was determined by GC-MS based on aryl halide using the normalized peak areas method. The Heck-Mizoroki coupling yields between styrene with phenyl iodide, 4-iodoanisole or 4-bromo-acetophenone were also determined as an isolated yield for the comparison purposes with the GC based conversion (Table 2, entries 12–16, 22–26 and 32–36). The isolated yields were determined as follow: at the end of the coupling reaction, the mixture was cooled to room temperature; the contents of the reaction vessel were poured into a separatory funnel. Water (3 mL) and ethyl acetate (5 mL) were added, and the coupling product was extracted and removed. After further extraction of the aqueous phase with ethyl acetate (5 mL) and combining the extracts, the ethyl acetate was removed *in vacuo* leaving the *trans*-stilbene or corresponding derivatives which was characterized by comparison of NMR data with that in the literature. 

### 3.3. General Procedure for the Suzuki Reactions

Pd(OAc)_2_ (1 mmol %), benzimidazolium halides **1**–**10** (2 mmol %), aryl halide (1 mmol), phenylboronic acid (1.2 mmol), K_2_CO_3_ (2 mmol), water (3 mL), DMF (3 mL) were added to microwave apparatus and the mixture was heated at 80 °C (300 W) for 10 min. A ramp time of 3 min was used to reach the temperature of 80 °C. At the end of reaction, the mixture was cooled, the product extracted with ethyl acetate/*n*-hexane (1:5), chromatographed on a silica gel column. The purity of coupling products was checked by NMR and GC-MS, and yields are based on aryl halide. The coupling products were confirmed by increasing the peaks on gas chromatograms and mass values from MS spectrums. All coupling products were also isolated and characterized by ^1^H-NMR or MS before the serial catalytic work up each time. The Suzuki coupling yields between phenylboronic acid and phenyl iodide, 4-iodoanisole or 4-bromoacetophenone were also determined as an isolated yield for comparison purposes with the GC based yields (Table 4 entries, 12–16, 22–26 and 32–36). The isolated yields were determined as follows: at the end of the Suzuki coupling reaction, the mixture was cooled to room temperature, the contents of the reaction vessel were poured into a separatory funnel. Water (3 mL) and ethyl acetate (5 mL) were added, and the coupling product was extracted and removed. After further extraction of the aqueous phase with ethyl acetate (5 mL) and combining the extracts, the ethyl acetate was removed *in vacuo* leaving the coupling product which was characterized by comparison of NMR data with that in the literature. 

## 4. Conclusions

We have prepared ten non-symmetric 1,3-disubstituted benzimidazolium salts **1**–**10** bearing on the nitrogen atoms of azolium ring phenyl, 4-chlorophenyl, 3-cyanopropyl, 2-vinyloxyethyl, allyl and 4-chlorobenzyl substituents. The catalytic activity of the novel benzimidazolium salts were evaluated using catalytic systems consisting of Pd(OAc)_2_/benzimidazolium salt and K_2_CO_3_ for the Heck-Mizoroki and Suzuki-Miyaura cross coupling reactions. The catalyst systems in the Heck-Mizoroki and Suzuki-Miyaura reactions gave better yields under microwave-assisted moderate conditions after very short reaction times compared to those given in the literature [52,57,58,59]. In addition, coupling of 2-pyridyl bromide with both styrene and phenylboronic acid afford the corresponding stilbene and biaryl products in satisfactory yields after 10 min.

## Supplementary Materials

NMR spectra of the new compounds and some coupling products are available free of charge via the internet at http://www.mdpi.com/1420-3049/18/3/2501/s1.
