# Supplementary material for: Synthesis, Characterization and Microwave-Promoted Catalytic Activity of Novel N-phenylbenzimidazolium Salts in Heck-Mizoroki and Suzuki-Miyaura Cross-Coupling Reactions under Mild Conditions
Source: Molecules. 2013 Feb 25;18(3):2501–17. doi: 10.3390/molecules18032501 (PMC6269800; doi:10.3390/molecules18032501)

Supplementary Materials

Table of Contents

NMR Spectra benzimidazolium salts and some coupling products. **S2–S13**

Figure S1. ^{1}H-NMR spectrum of compound **1**.

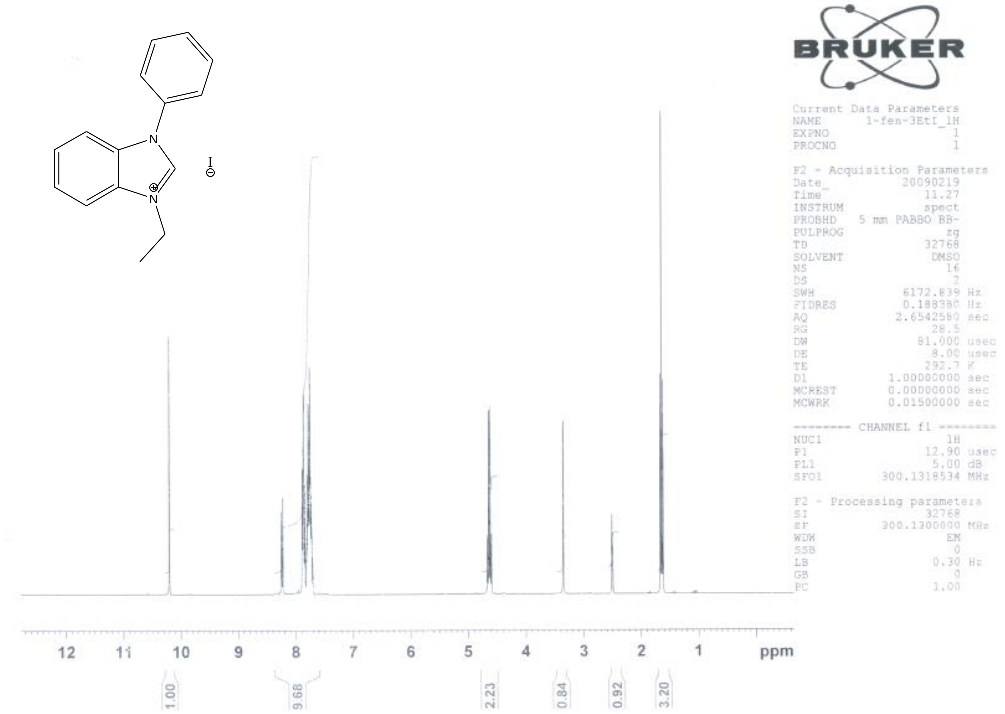

Figure S2. ^{13}C-NMR spectrum of compound **1**.

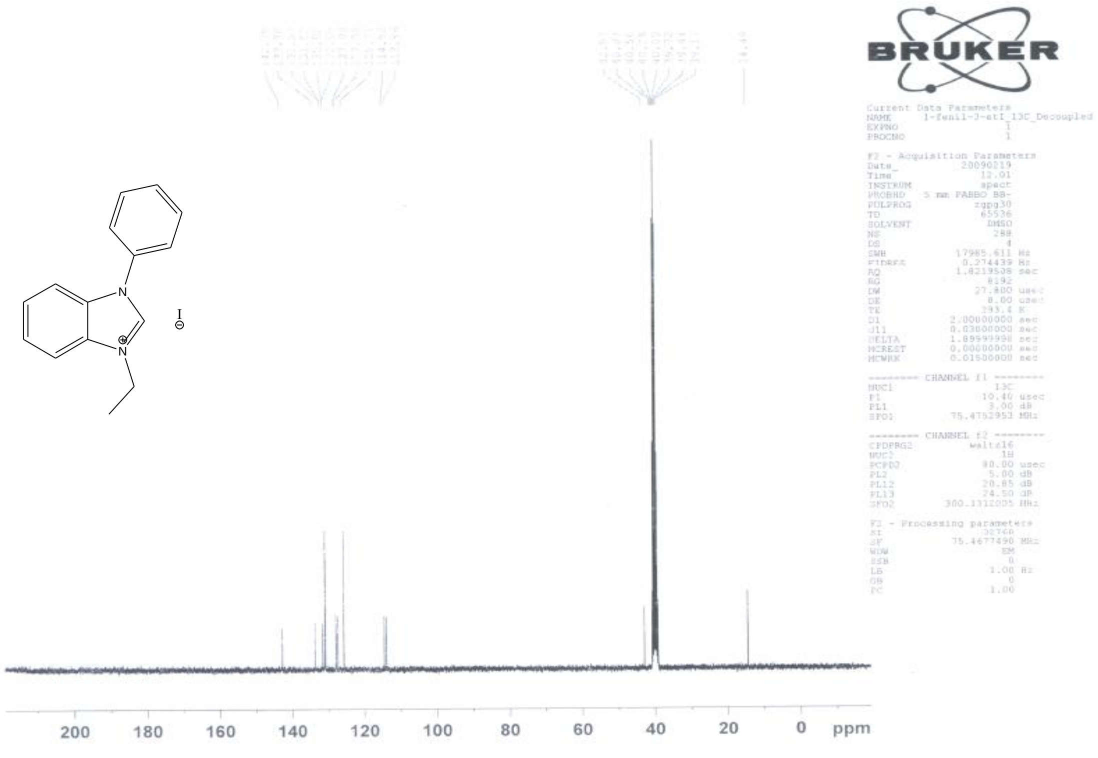

Figure S3. ^{1}H-NMR spectrum of compound **2**.

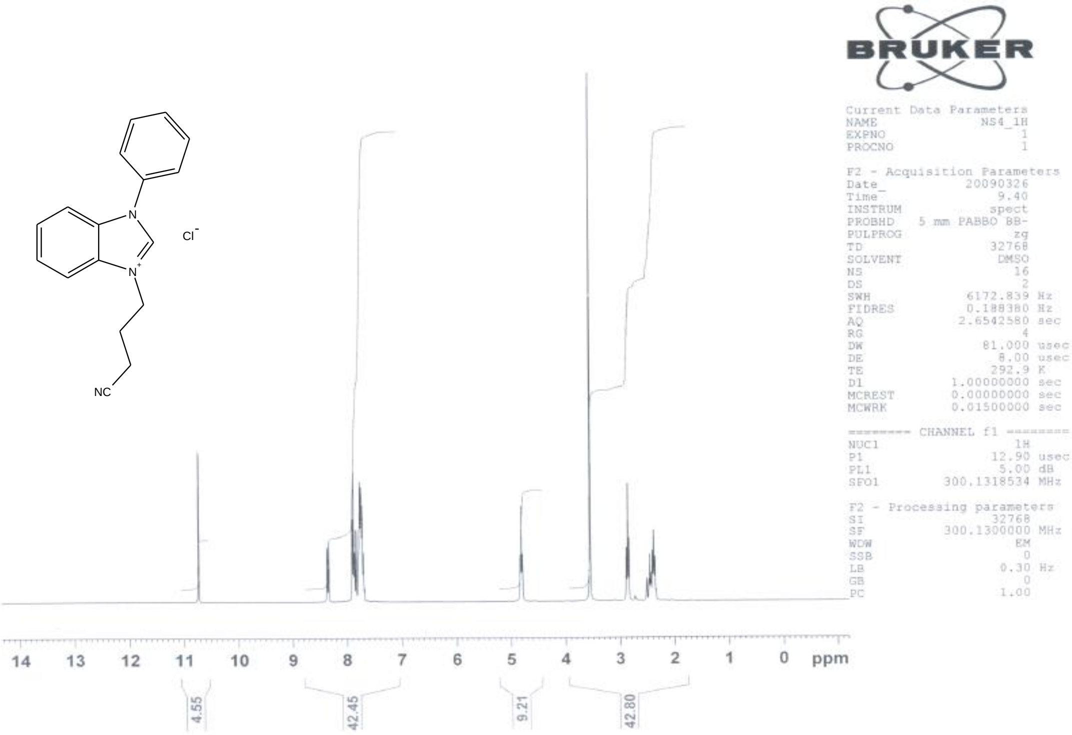

Figure S4. ^{13}C-NMR spectrum of compound **2**.

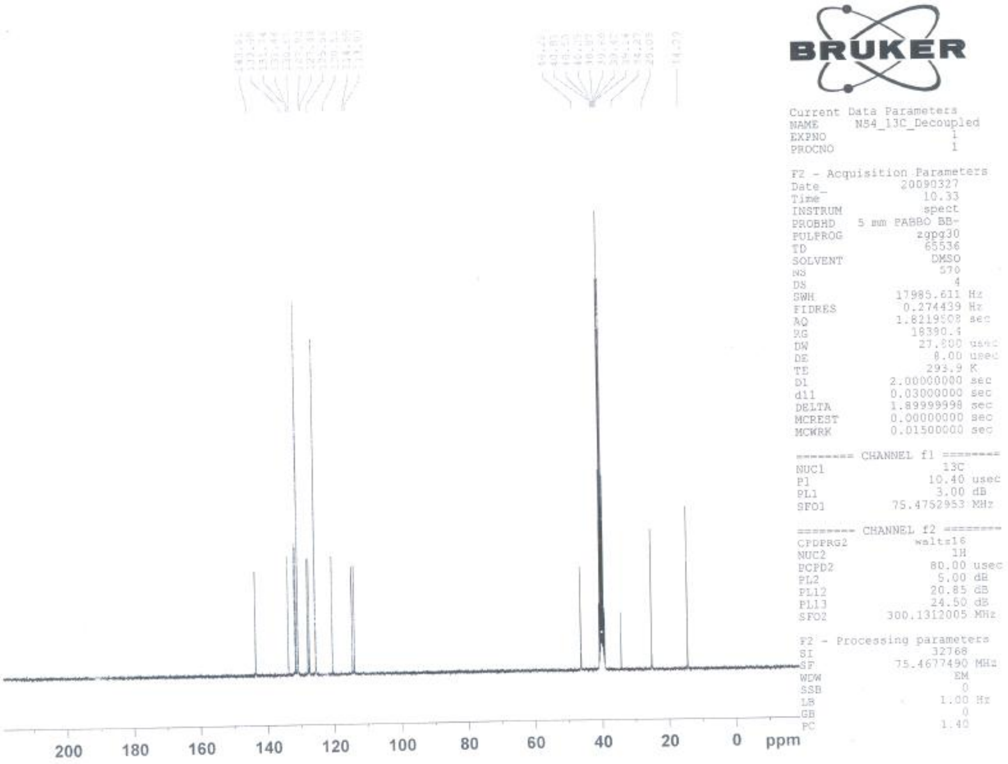

Figure S5. ^{1}H-NMR spectrum of compound **3**.

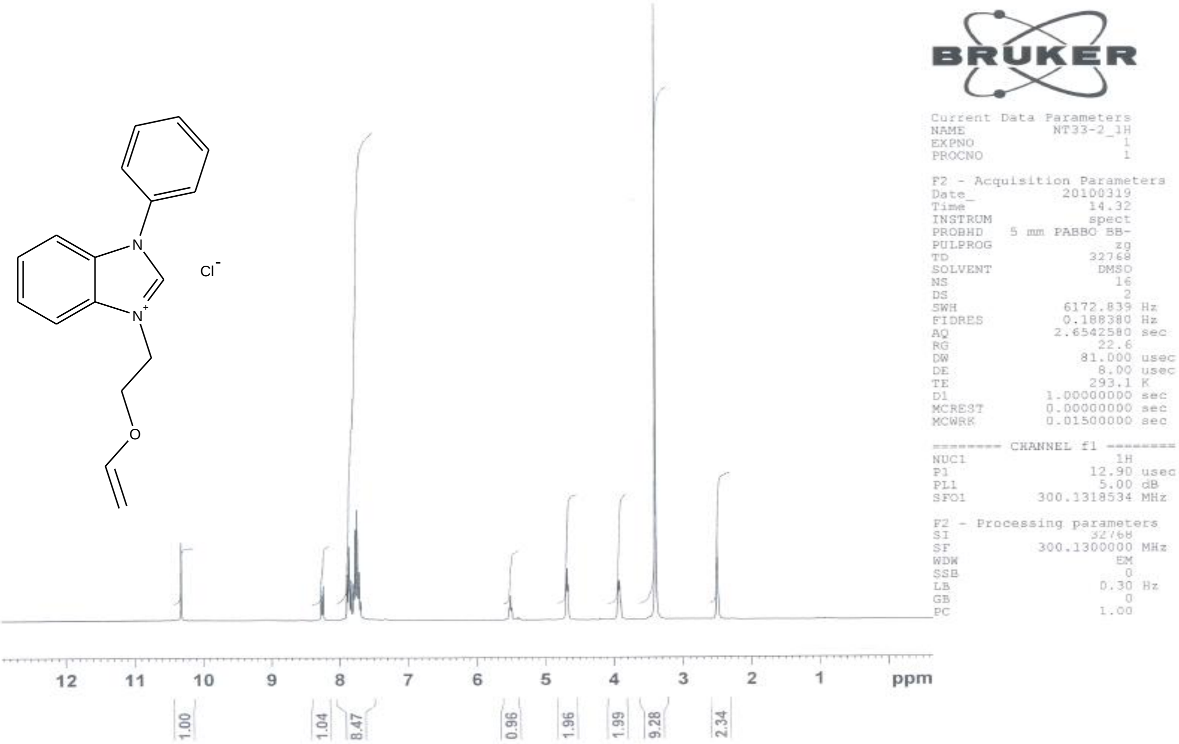

Figure S6. ^{13}C-NMR spectrum of compound **3**.

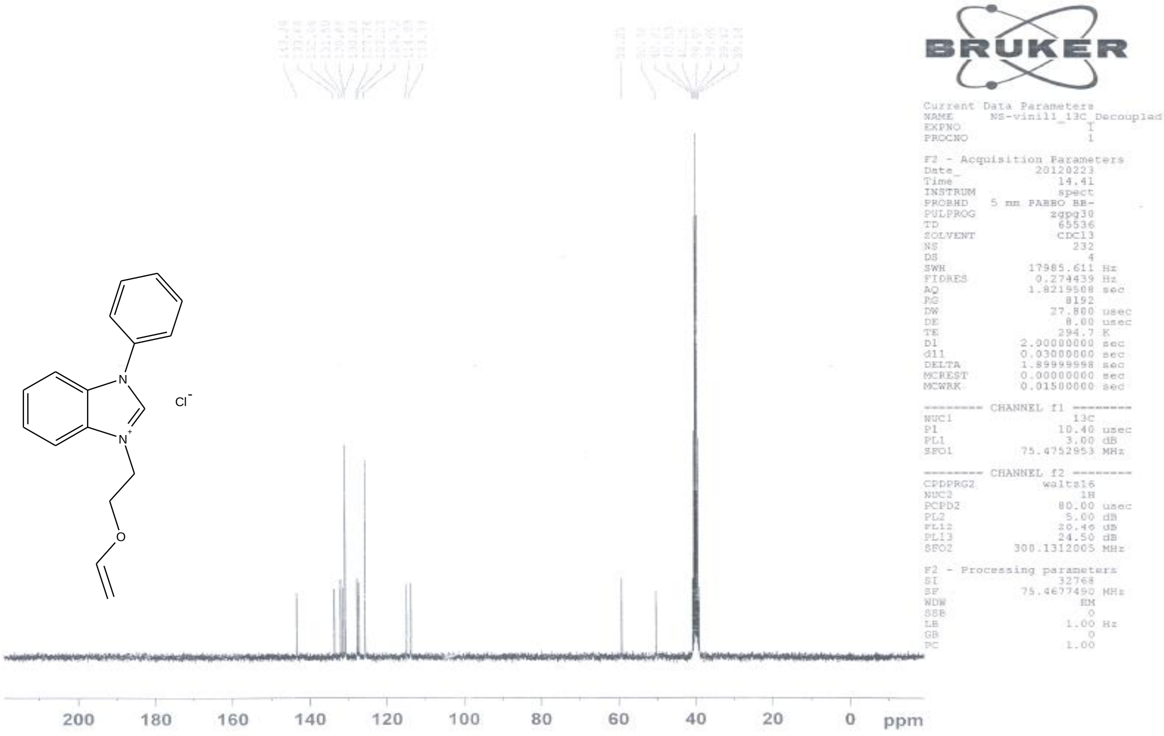

Figure S7. ^{1}H-NMR spectrum of compound **4**.

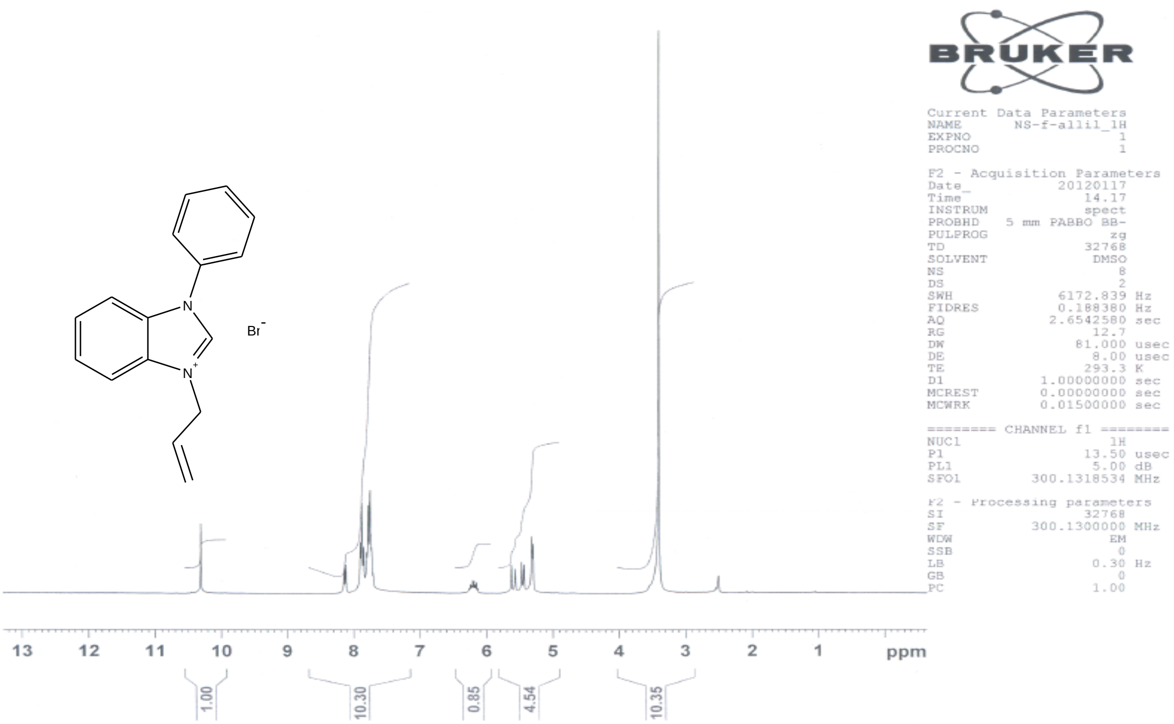

Figure S8. ^{13}C-NMR spectrum of compound **4**.

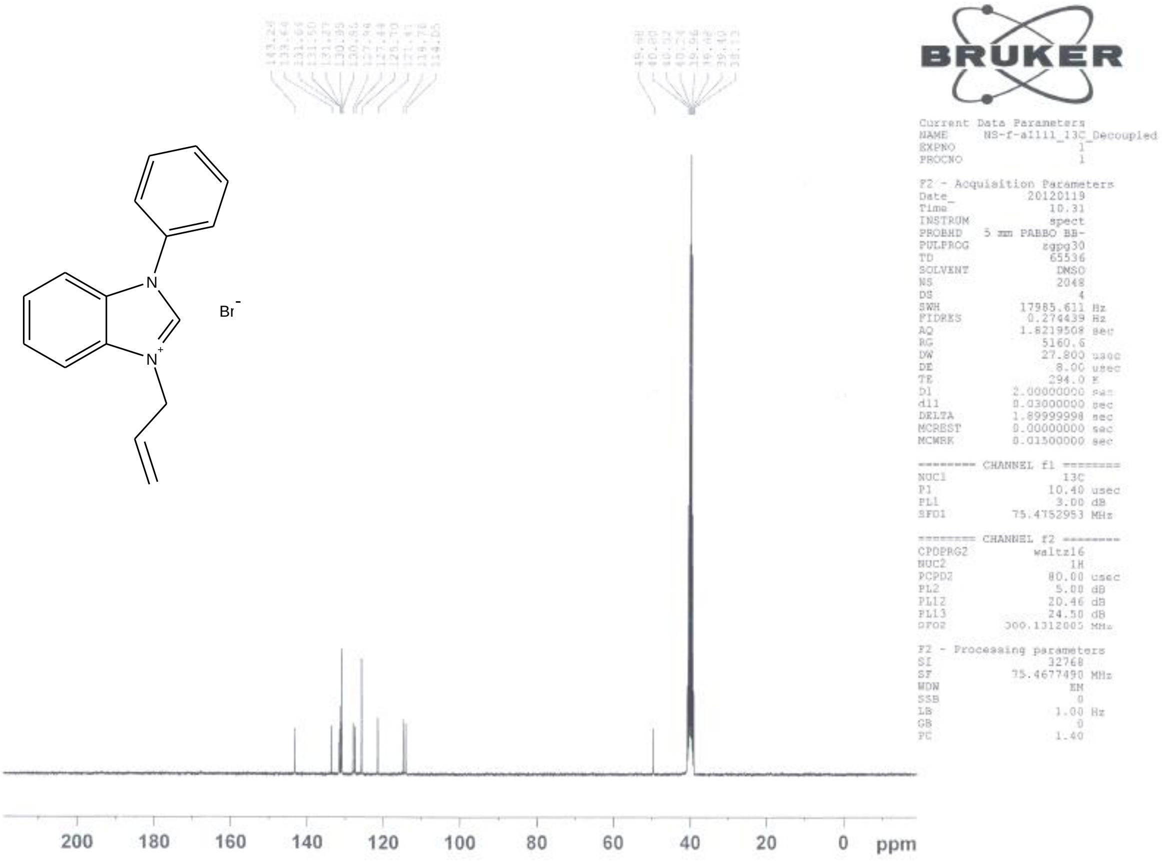

Figure S9. ^{1}H-NMR spectrum of compound **5**.

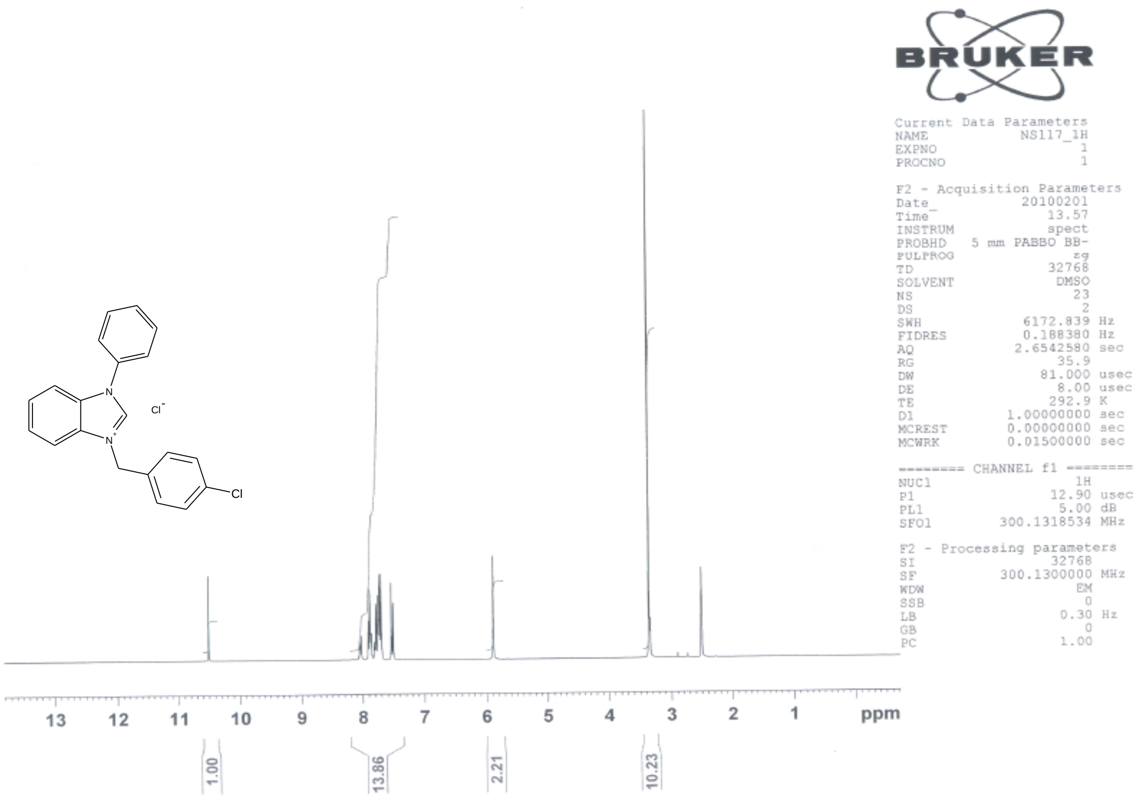

Figure S10. ^{13}C-NMR spectrum of compound **5**.

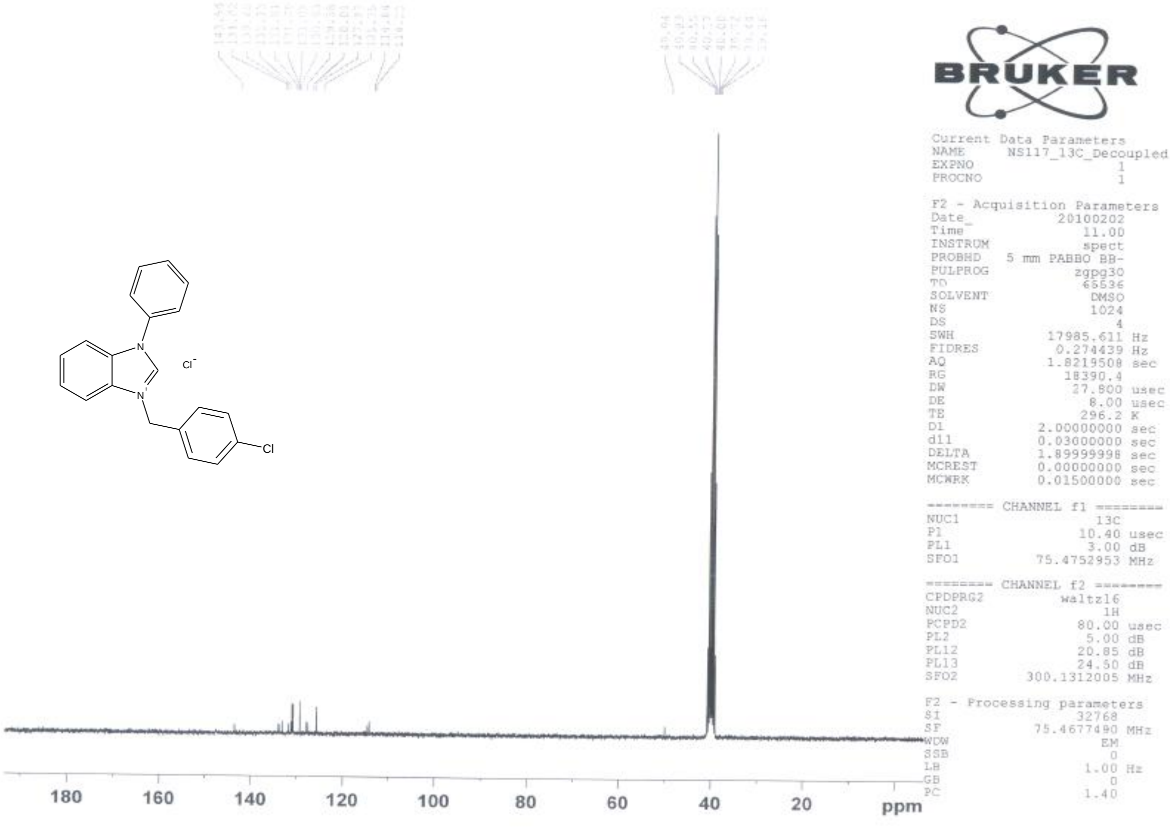

Figure S11. ^{1}H-NMR spectrum of compound **6**.

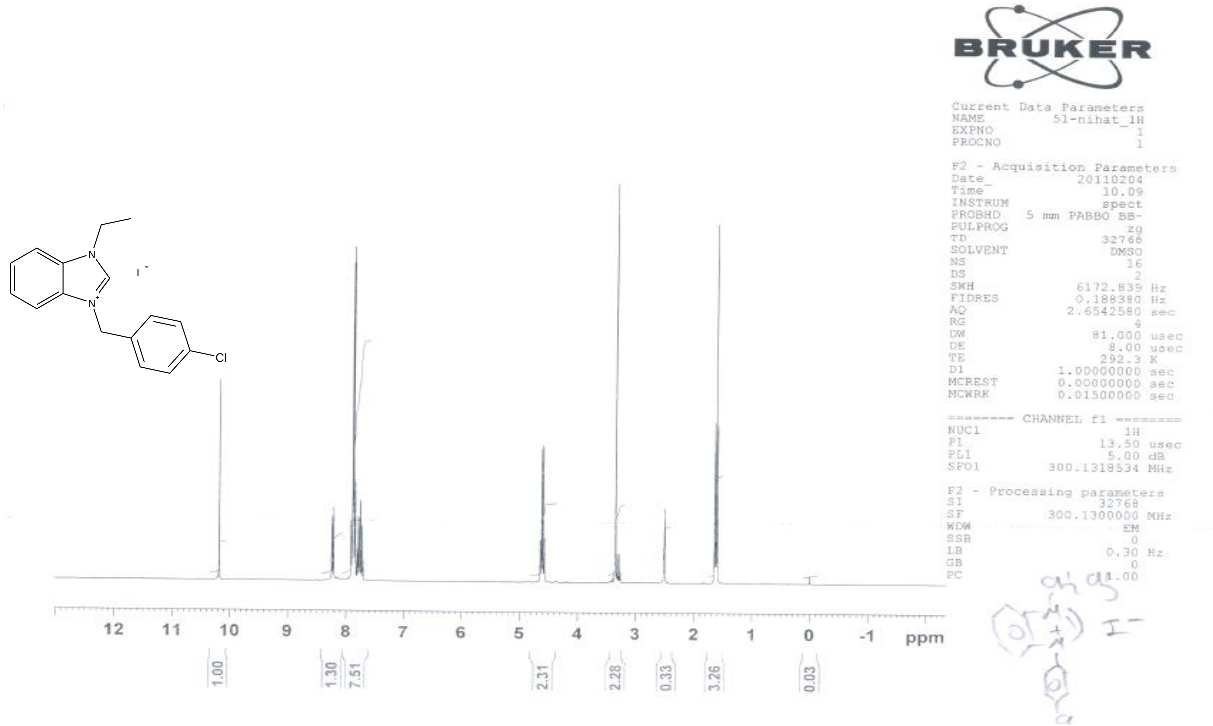

Figure S12. ^{13}C-NMR spectrum of compound **6**.

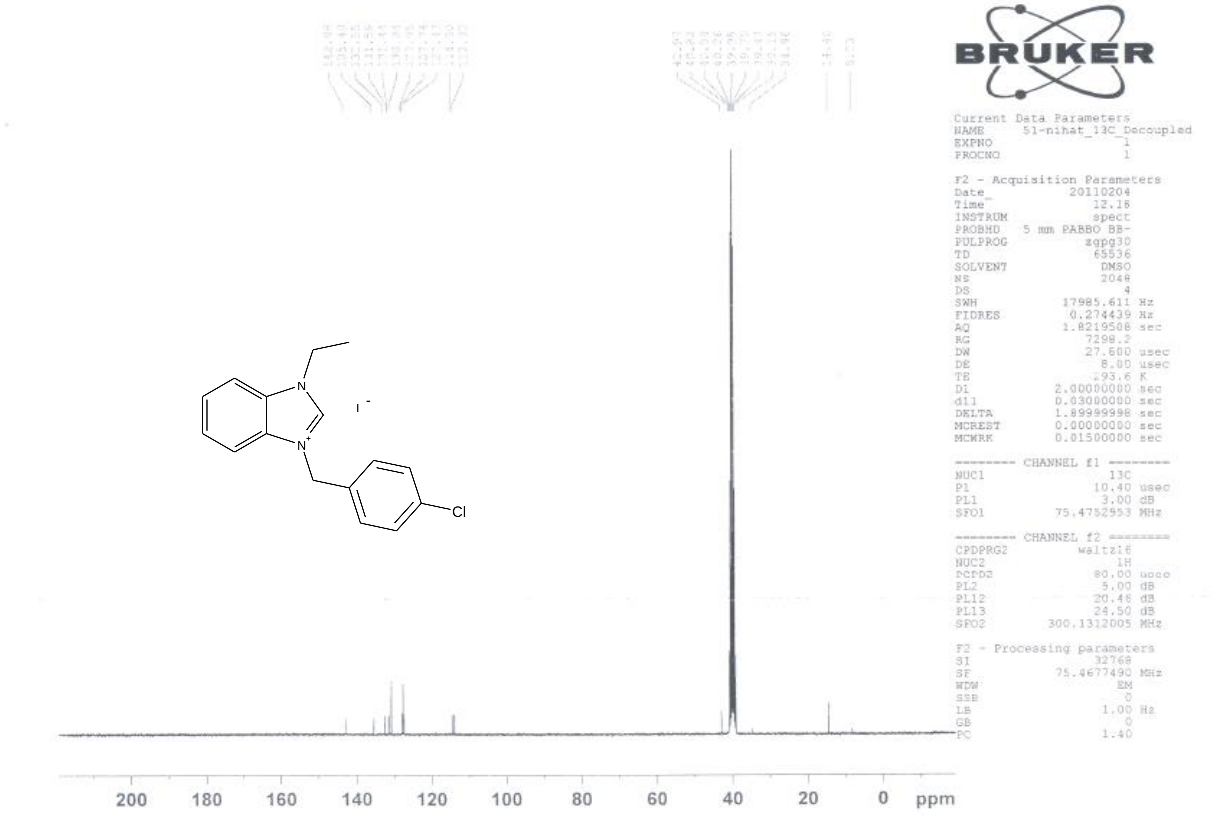

Figure S13. ^{1}H-NMR spectrum of compound **7**.

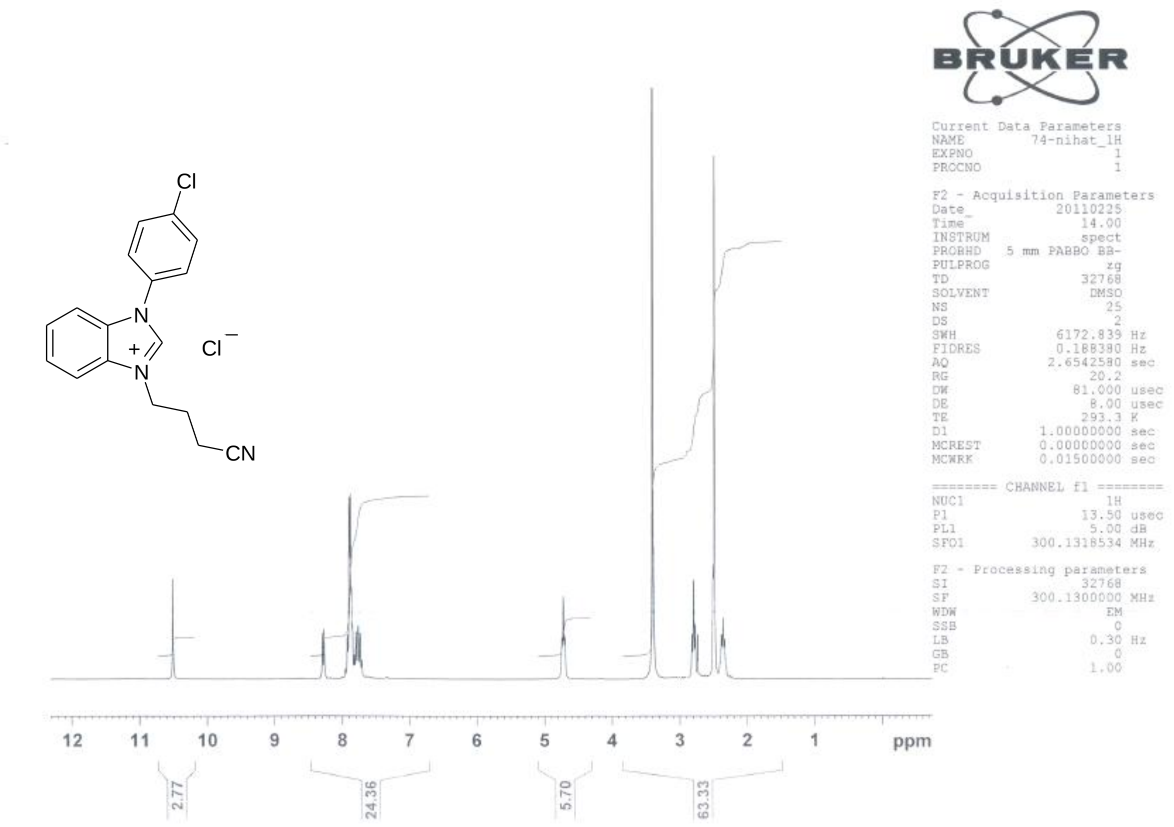

Figure S14. ^{13}C-NMR spectrum of compound **7**.

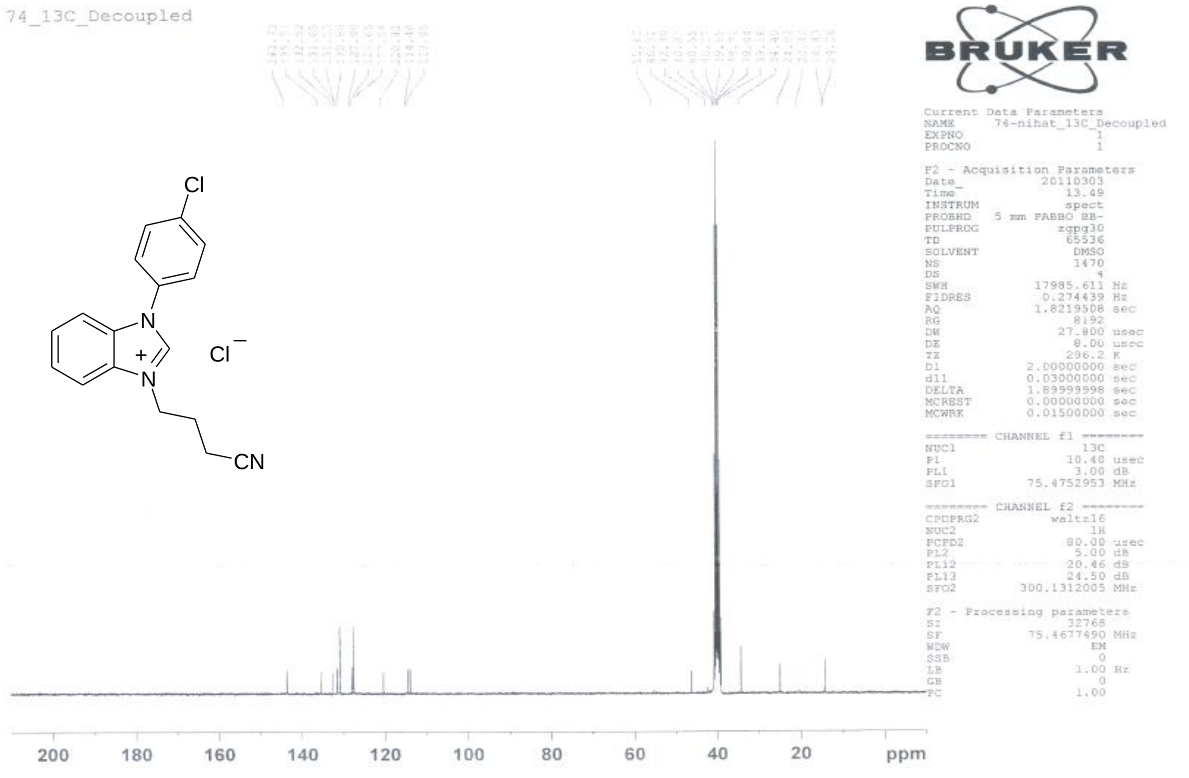

Figure S15. ^{1}H-NMR spectrum of compound **8**.

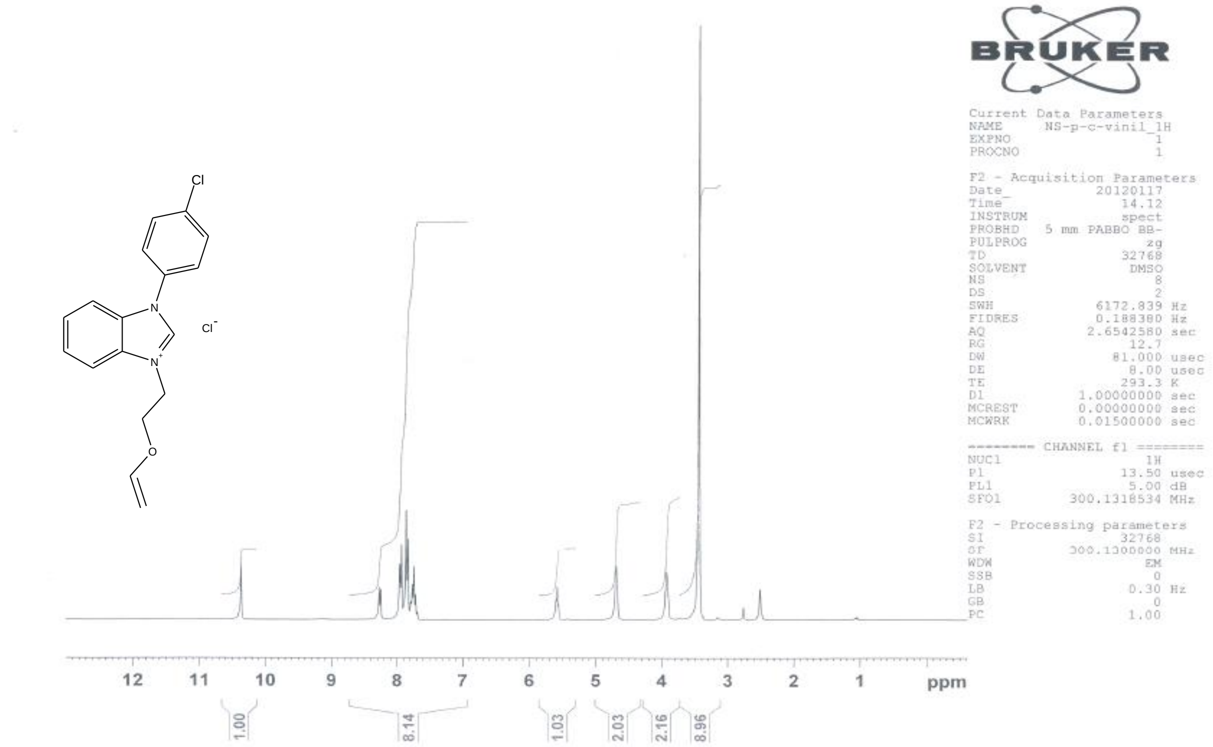

Figure S16. ^{13}C-NMR spectrum of compound **8**.

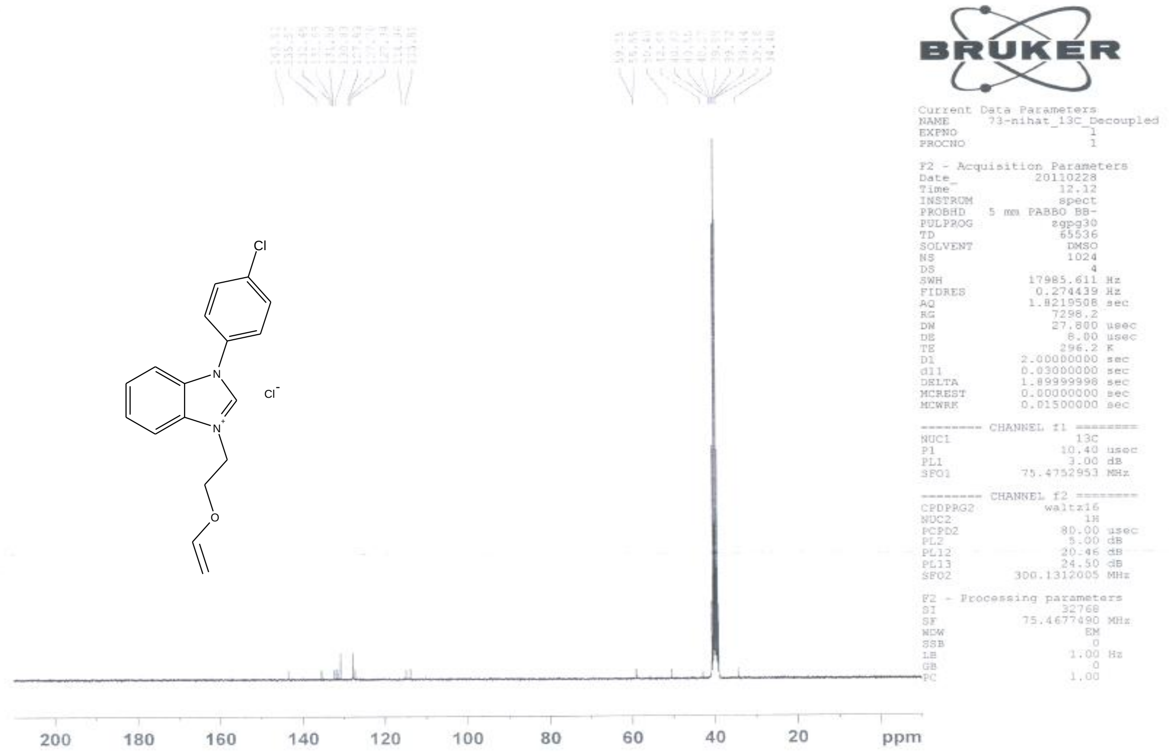

Figure S17. ^{1}H-NMR spectrum of compound **9**.

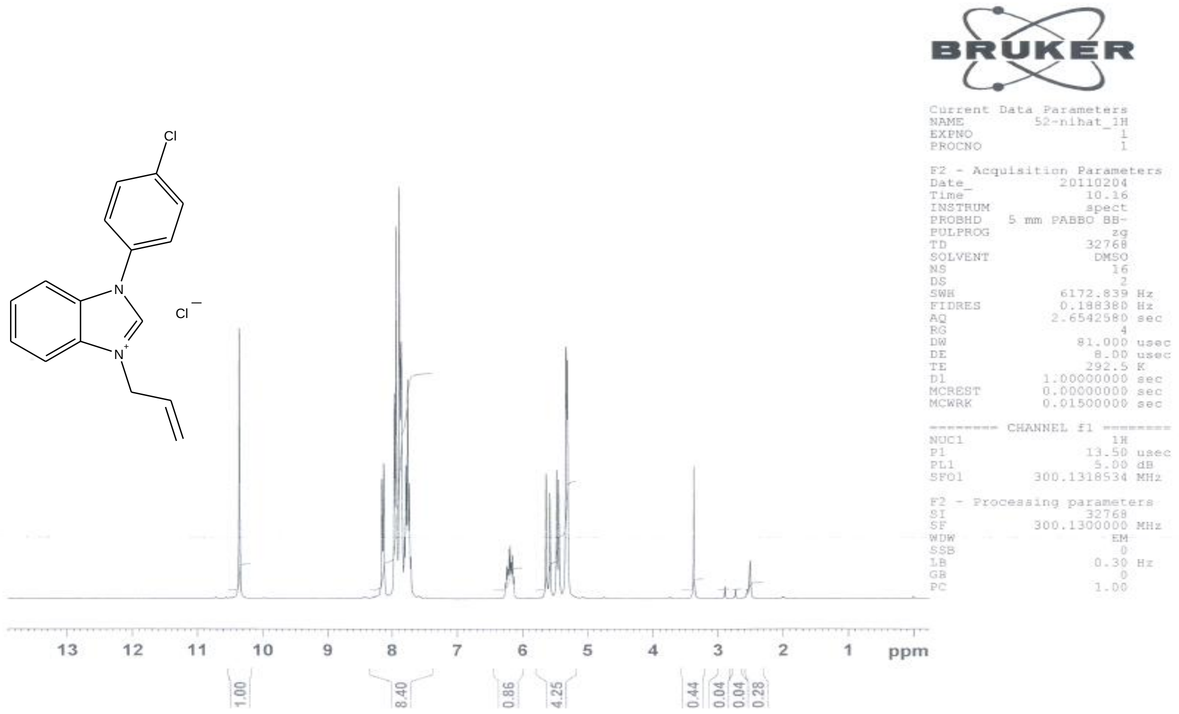

Figure S18. ^{13}C-NMR spectrum of compound **9**.

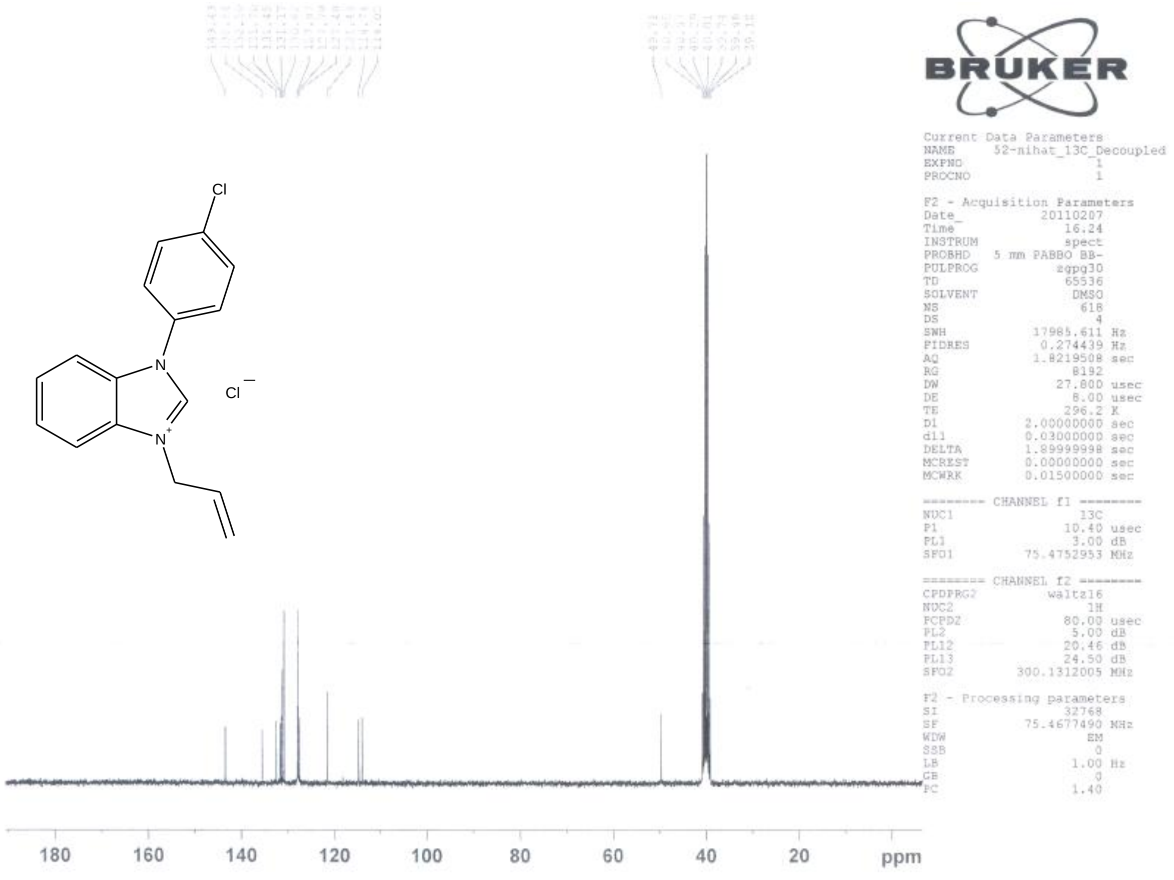

Figure S19. ^{1}H-NMR spectrum of compound **10**.

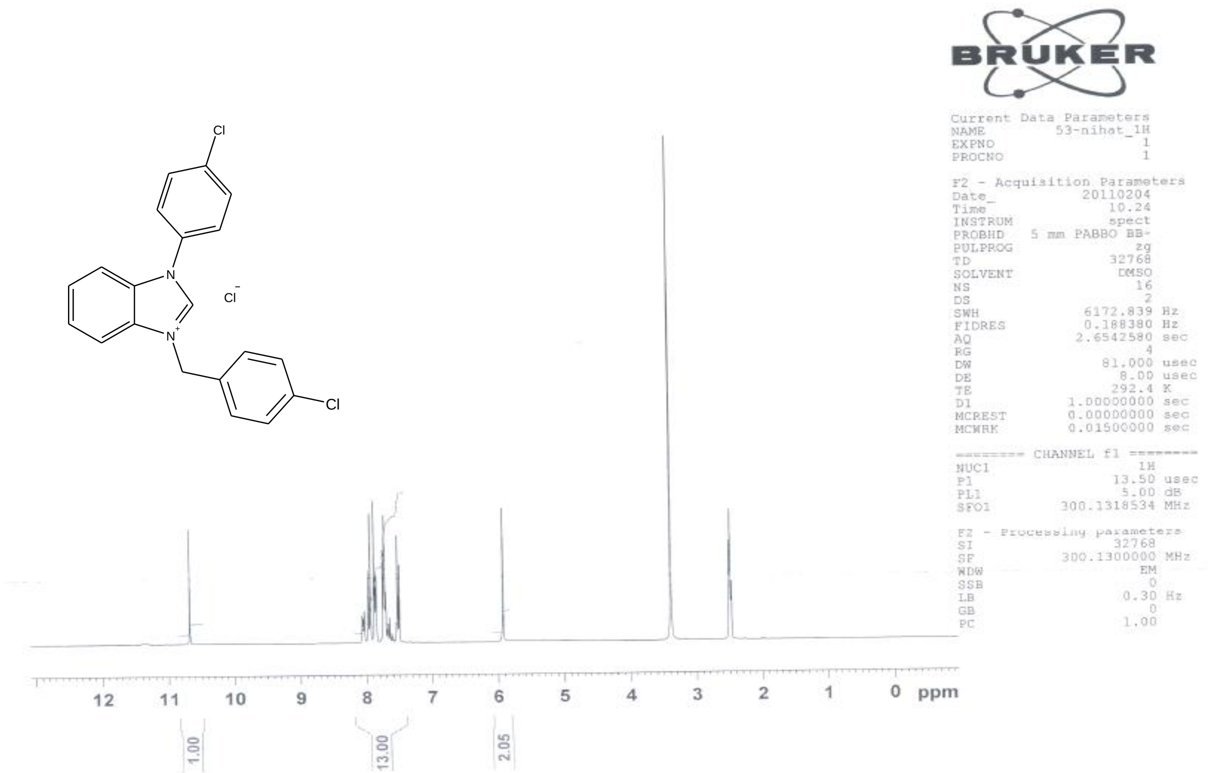

Figure S20. ^{13}C-NMR spectrum of compound **10**.

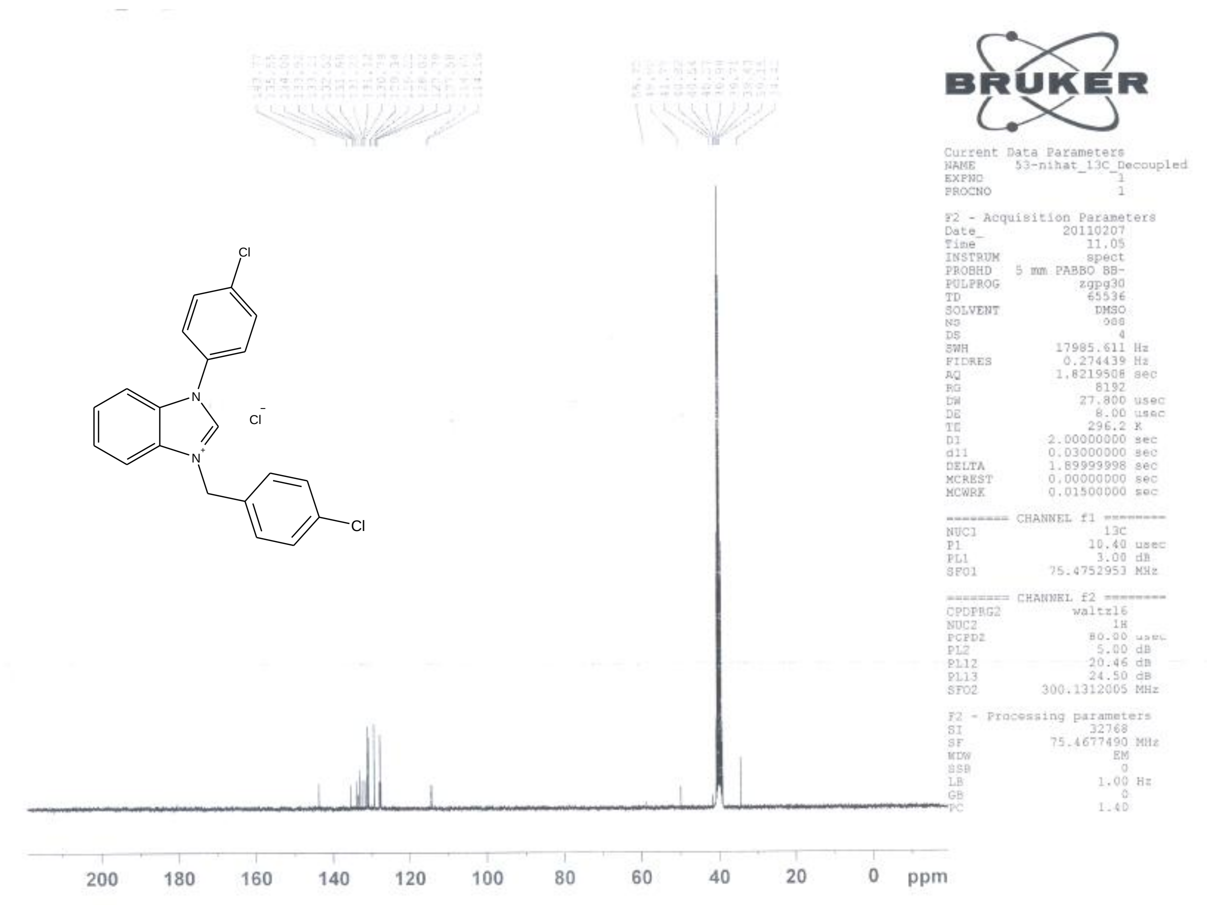

Figure S21. ^{1}H-NMR spectrum of biphenyl.

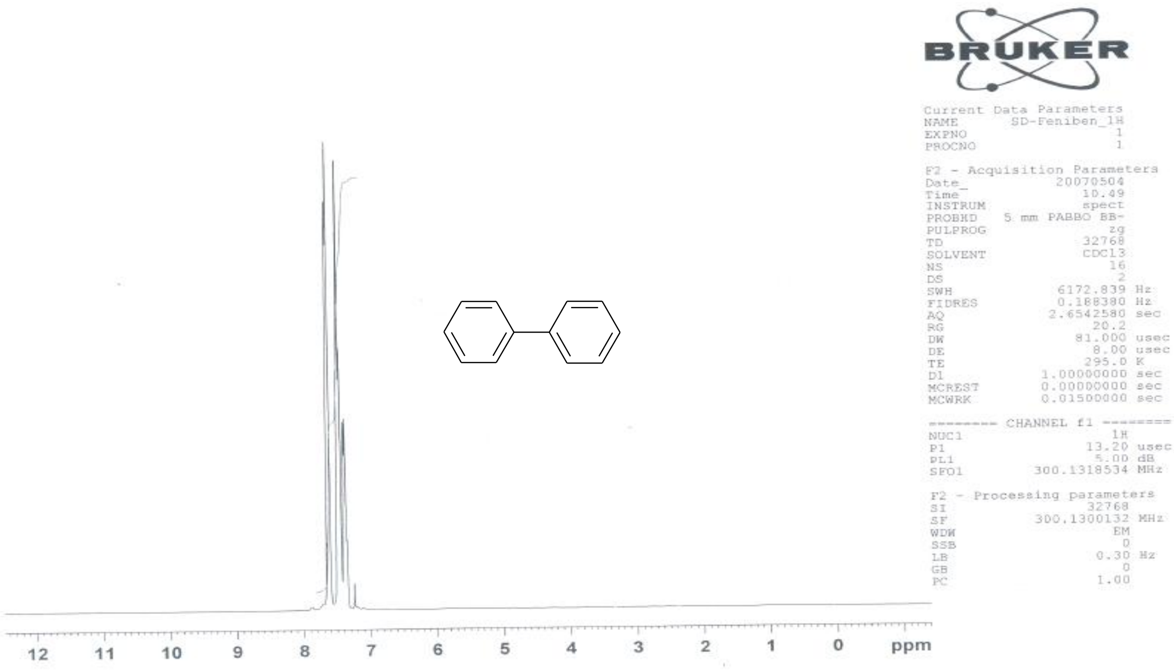

Figure S22. ^{1}H-NMR spectrum of 4-methoxybiphenyl.

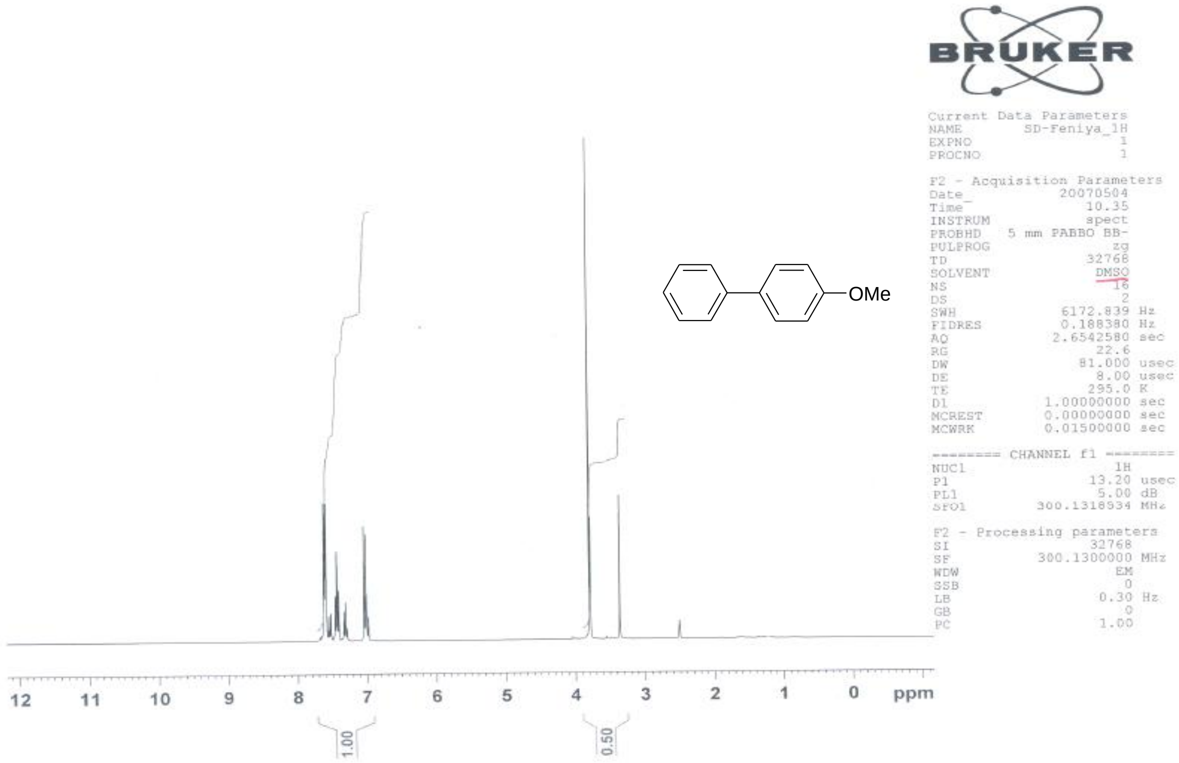

Figure S23. ^{1}H-NMR spectrum of 4-acetylbiphenyl.

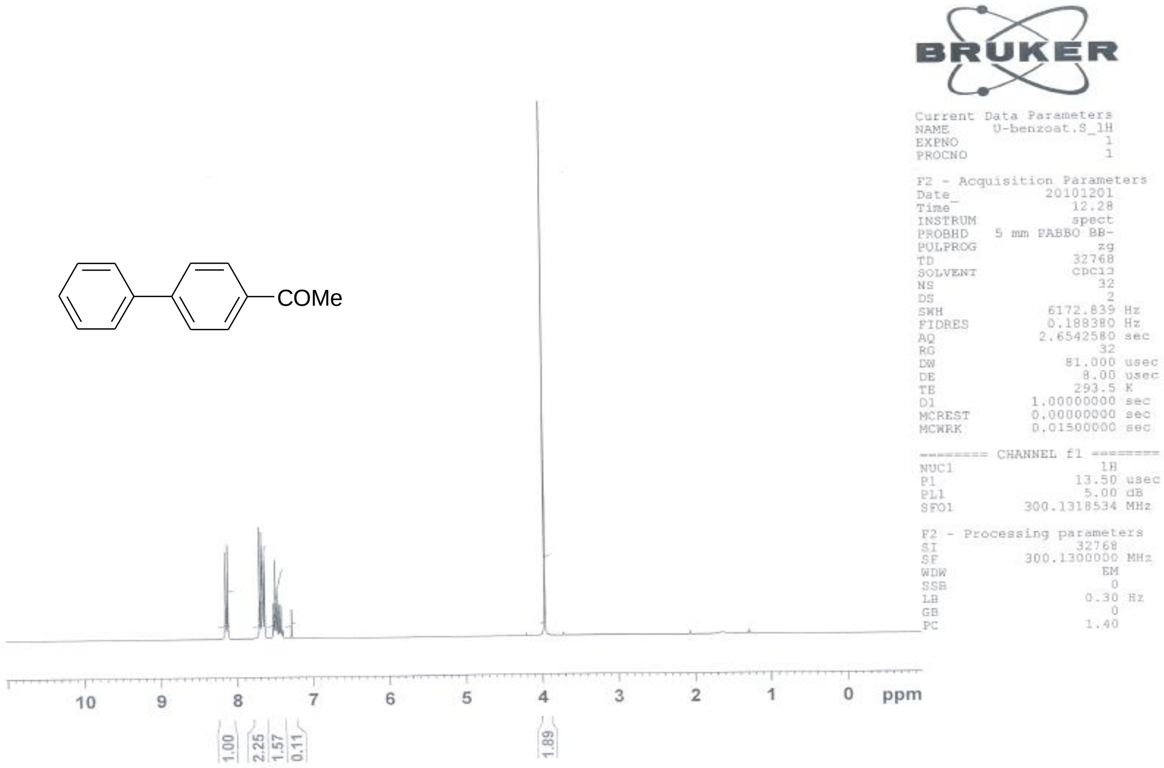

Figure S24. ^{1}H-NMR spectrum of *trans*-stilbene.

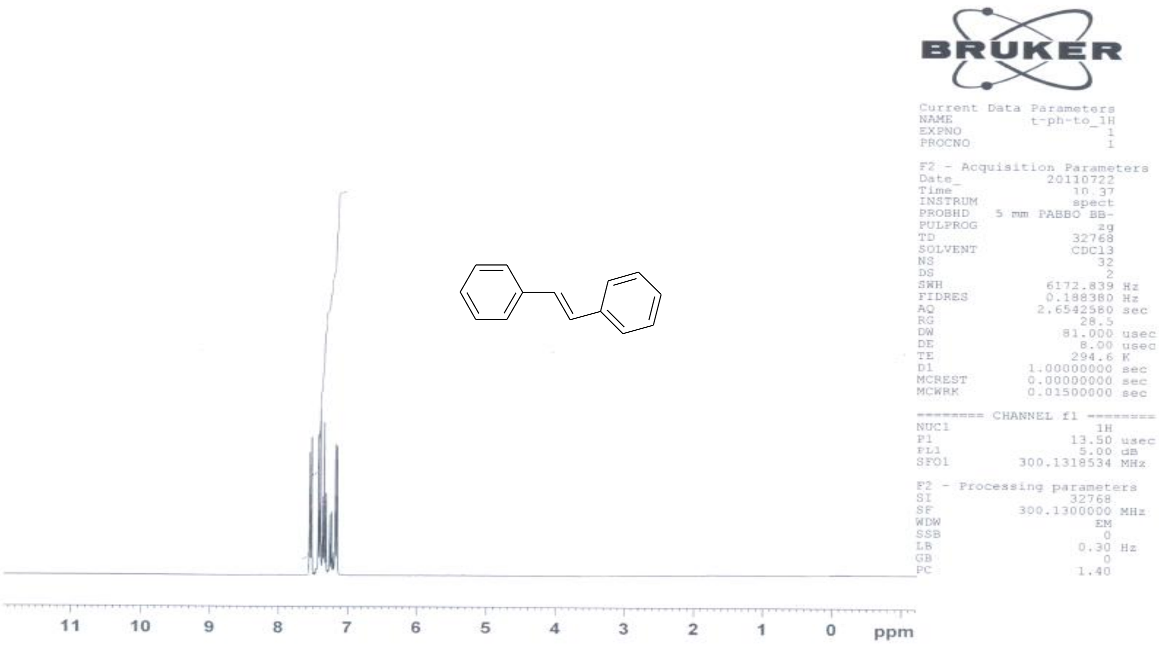

Figure S25. ¹H-NMR spectrum of *trans*-4-methoxystilbene.

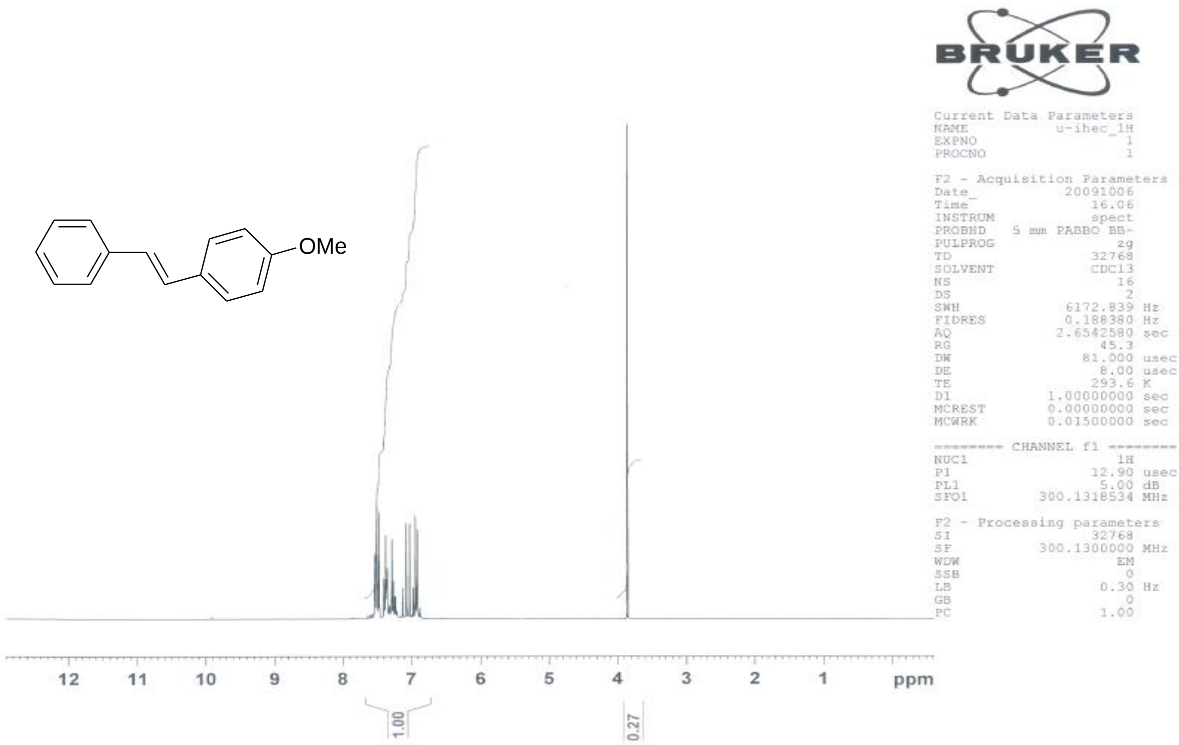

Figure S26. ¹H-NMR spectrum of *trans*-4-acetylstilbene.

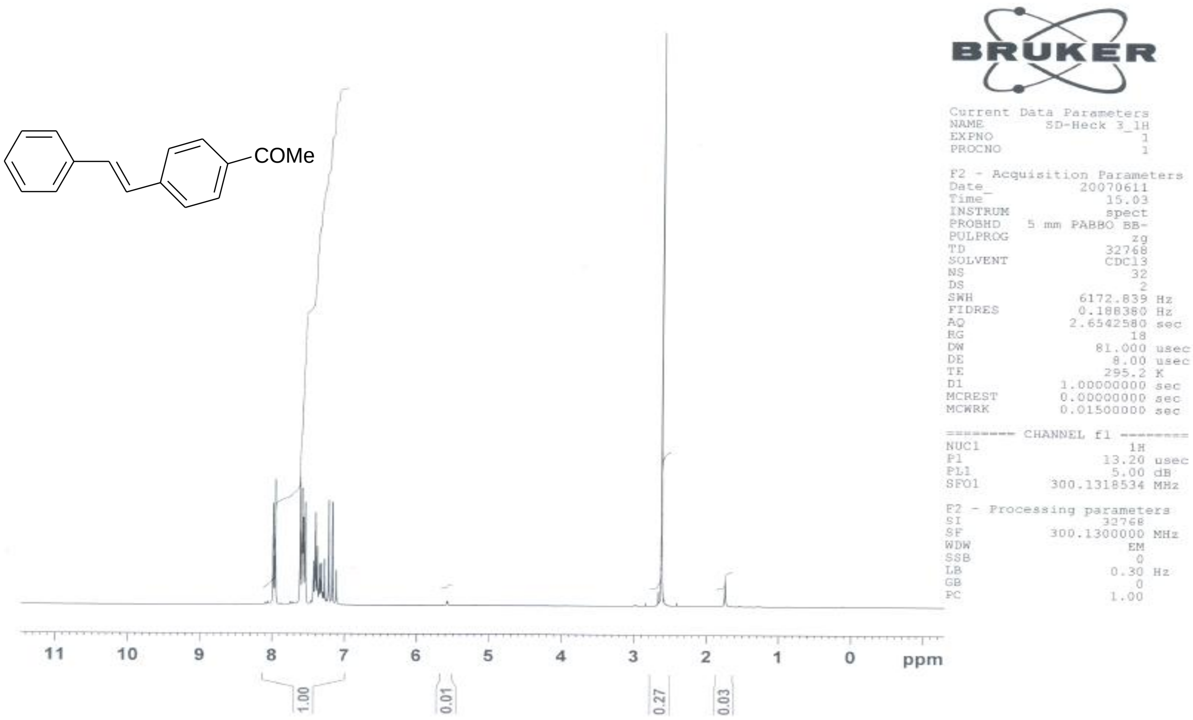

Supplement: Supplementary file 1 [file molecules-18-02501-s001.pdf]
